# Mieap, a p53-Inducible Protein, Controls Mitochondrial Quality by Repairing or Eliminating Unhealthy Mitochondria

**DOI:** 10.1371/journal.pone.0016060

**Published:** 2011-01-17

**Authors:** Noriaki Kitamura, Yasuyuki Nakamura, Yuji Miyamoto, Takafumi Miyamoto, Koki Kabu, Masaki Yoshida, Manabu Futamura, Shizuko Ichinose, Hirofumi Arakawa

**Affiliations:** 1 Cancer Medicine and Biophysics Division, National Cancer Center Research Institute, Tokyo, Japan; 2 Instrumental Analysis Research Center, Tokyo Medical and Dental University, Tokyo, Japan; University of Medicine and Dentistry of New Jersey, United States of America

## Abstract

Maintenance of healthy mitochondria prevents aging, cancer, and a variety of degenerative diseases that are due to the result of defective mitochondrial quality control (MQC). Recently, we discovered a novel mechanism for MQC, in which Mieap induces intramitochondrial lysosome-like organella that plays a critical role in the elimination of oxidized mitochondrial proteins (designated MALM for Mieap-induced accumulation of lysosome-like organelles within mitochondria). However, a large part of the mechanisms for MQC remains unknown. Here, we report additional mechanisms for Mieap-regulated MQC. Reactive oxygen species (ROS) scavengers completely inhibited MALM. A mitochondrial outer membrane protein NIX interacted with Mieap in a ROS-dependent manner via the BH3 domain of NIX and the coiled-coil domain of Mieap. Deficiency of NIX also completely impaired MALM. When MALM was inhibited, Mieap induced vacuole-like structures (designated as MIV for Mieap-induced vacuole), which engulfed and degraded the unhealthy mitochondria by accumulating lysosomes. The inactivation of p53 severely impaired both MALM and MIV generation, leading to accumulation of unhealthy mitochondria. These results suggest that (1) mitochondrial ROS and NIX are essential factors for MALM, (2) MIV is a novel mechanism for lysosomal degradation of mitochondria, and (3) the p53-Mieap pathway plays a pivotal role in MQC by repairing or eliminating unhealthy mitochondria via MALM or MIV generation, respectively.

## Introduction

Mitochondria are essential to oxidative energy production in aerobic eukaryotic cells and are required for multiple biosynthetic pathways [1]. Therefore, mitochondrial quality control (MQC) is of significant importance for maintaining the steady and healthy state of our bodies [2]. Dysregulation of MQC causes various phenomena and diseases including aging, cancer, and degenerative diseases [3]. However, mechanisms of MQC have not been fully elucidated. Currently, two possible mechanisms are suggested. The first one is lysosomal degradation of the entire mitochondrion, known as mitophagy, which is mediated by double-membraned autophagosomes [4], [5]. The second one is protease-dependent degradation of damaged proteins within mitochondria, which also plays a pivotal role in maintaining the healthy status of mitochondria [6]. Previous studies have reported the existence of several mitochondrial proteases including LON and AAA proteases, which play a critical role in degradation of mitochondrial proteins [7]. In addition to these two mechanisms, we discovered a third mechanism for MQC, in which Mieap, a p53-inducible protein, induces intramitochondrial lysosome-like organella without destroying the mitochondrial structure (designated MALM for Mieap-induced accumulation of lysosome-like organelles within mitochondria), leading to the elimination of oxidized mitochondrial proteins and improvement of mitochondrial functions [8]. Interestingly, the mechanism was completely different from canonical autophagy [8]. Although MALM seems to play a crucial role in maintenance of healthy mitochondria, a large part of the mechanism is still unknown.

NIX (also designated BNIP3L) is a BH3-domain protein that belongs to the Bcl-2 family [9]. The homologous protein BNIP3 shares 55% amino acid sequence similarity with NIX [10]. NIX is localized to the mitochondrial outer membrane and regulates cell death [11]. Interestingly, in contrast to other mitochondrial Bcl-2 family proteins, NIX and BNIP3 are not involved in the release of cytochrome c and the resulting caspase-dependent apoptosis, but rather related to necrosis through the regulation of mitochondrial permeability transition pore (MPTP) [12]. NIX was also shown to localize to endoplasmic reticulum (ER) and increase the store of Ca^++^, leading to Ca^++^ influx into mitochondria and cell death [13]. NIX was also shown to regulate canonical autophagy of mitochondria during the process of erythroid cell differentiation [14], [15]. Furthermore, the role of NIX and BNIP3 in hypoxia-induced autophagy was also reported [16]. Although many functions have been suggested, the physiological role of NIX remains unclear.

Degradation of the whole structure of mitochondrion is mediated by autophagosomes in mammalian cells, which is called as mitophagy [4]. Damaged mitochondria are initially sequestrated by double-membraned autophagosomes, which fuse to lysosomes, leading to mitochondrial degradation [17]. Very recently, NIX and Parkin/PINK1 were reported to mediate the process of mitophagy in erythroid cells [14], [15] and neuronal cells [18], [19], respectively. In that context, NIX may function as a mitochondrial receptor for autophagy by interacting with LC3 [20]. The Parkin/PINK1 pathway regulates ubiquitylation of p62 and VDAC, leading to final clearance of the damaged mitochondria by autophagosome-mediated autophagy [21]. Therefore, it is likely that double-membraned autophagosomes play an essential role in mitochondrial autophagy in mammalian cells. On the other hand, Mizushima et al. reported that mitochondrial degradation during the lens and erythroid differentiation occurred normally in macroautophagy-deficient ATG5^−/−^ mice, implying that there are alternative pathways of mitophagy [22].

In contrast to mammalian cells, the delivery of unhealthy mitochondria to cytoplasmic large vacuoles is the central mechanism for degradation of mitochondria in yeast cells [23]. Yeast cells usually contain large vacuoles in the cytoplasm, which are equivalent to lysosomes in mammalian cells [23]. There are two non-selective pathways for degradation of mitochondria in yeast (macroautophagy and microautophagy), in which the vacuole nonspecifically uptakes mitochondria with the surrounding cytosol [23]. In contrast, selective microautophagy of mitochondria specifically eliminates unhealthy mitochondria in yeast cells [24]; the mitochondrial outer membrane protein Uth1p plays a critical role in this process [25]. Interestingly, mitochondrial ROS and the ROS-modified mitochondrial outer membrane may play a critical role in targeting damaged mitochondria in yeast cells [26], whereas the depolarization of mitochondria was suggested to trigger mitophagy in mammalian cells [5]. Recently, ATG32, a mitochondrial outer membrane protein, was identified to mediate selective degradation of mitochondria in yeast cells [27], [28]. However, Uth1p and ATG32 do not have any sequence similarity with mammalian mitophagy-related proteins including NIX and Parkin, suggesting that there may be many differences in mitophagy between yeast and human.

Here, we report the role of NIX and ROS in MALM. Moreover, we identified an additional mechanism for Mieap-regulated MQC, in which the Mieap-induced vacuole (designated MIV) eats unhealthy mitochondria, representing a novel mechanism for mitophagy in mammalian cells. Furthermore, we demonstrated that p53 and/or Mieap plays a pivotal role in MQC by repairing or eliminating unhealthy mitochondria via MALM or MIV generation, respectively.

## Results

### Mitochondrial ROS play a key role for discriminating the unhealthy mitochondria in MALM

**Figure 1:**
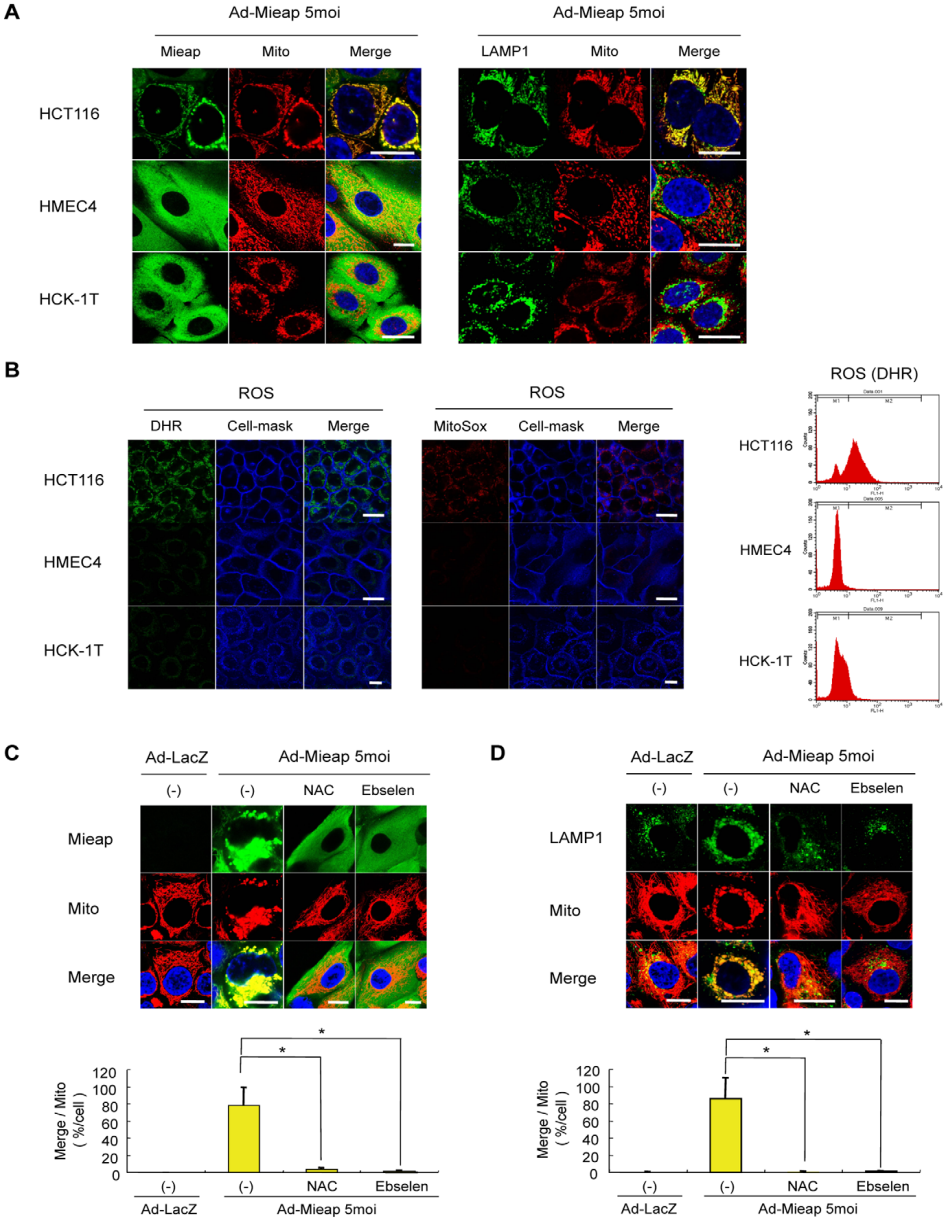
ROS plays a key role in Mieap-targeting the unhealthy mitochondria. (A) Exogenous Mieap does not affect normal cell mitochondria. One cancer cell line (HCT116: colorectal cancer cells), and two normal human cell lines (HMEC4: human mammary epithelial cells, and HCK-1T: human cervical keratinocyte cells) were infected with Ad-Mieap, and 3 days after infection, IF experiment was carried out. Mieap protein was stained with polyclonal rabbit anti-Mieap antibody (green). Lysosomes were stained with anti-LAMP1 antibody (green). Mitochondria were indicated by the DsRed-mito protein signal (red). Scale bar  = 20 µm. (B) Normal cell mitochondria produce much less ROS. The ROS level was detected in live cells of HCT116, HMEC4 and HCK-1T by MitoSox-red (red) and DHR (green). A representative image in each cell is shown. The ROS level was also analysed with DHR by FACS. Scale bar  = 20 µm. (C) ROS scavenger inhibits mitochondrial localization and accumulation of Mieap. HCT116 Ad-LacZ-infected and Ad-Mieap-infected cells of HCT116 were γ-ray-irradiated, and 3 days after IR, in the presence or absence of the ROS scavengers NAC and Ebselen, the cells were subjected to IF analysis. Mieap protein was stained with anti-Mieap antibody (green). Mitochondria were indicated by the DsRed-mito protein signal (red). (D) ROS scavenger inhibits MALM. Using the protocol described in (C), lysosomes were stained with anti-LAMP1 antibody (green), and mitochondria were indicated by the DsRed-mito protein signal (red). (C) (D) The yellow area indicated mitochondrial localization and accumulation of Mieap (C) or accumulation of lysosomes into mitochondria (D). Quantitative analysis of the yellow or red area was carried out in 300–400 cells. Average values for the ratio of yellow to red (merged/mitochondrial; yellow bar graph) are shown with error bars indicating 1 SD. p<0.01 (*) was considered statistically significant in the Ad-Mieap-infected cells with and without NAC or Ebselen. Scale bar  = 20 µm.

We recently discovered a novel mechanism for mitochondrial quality control, in which Mieap induces the intramitochondrial lysosomes that are involved in the elimination of oxidized mitochondrial proteins [8]. However, there are many questions that remain to be elucidated. One of the important questions is what are the underlying mechanisms involved in Mieap's ability to differentiate between healthy (containing normal proteins) and unhealthy (containing damaged and oxidized proteins) mitochondria. To further characterize the mechanisms involved in MALM, the effects of Mieap on normal cell mitochondria using HMEC4 and HCK-1T cells were examined. Interestingly, although Mieap induced MALM in HCT116 cancer cells, Mieap failed to recognize the normal cell mitochondria or induce accumulation of lysosomes in mitochondria (Figure 1A). Moreover, the ROS levels generated by the normal cell mitochondria were significantly much lower than those generated by the cancer cell mitochondria (Figure 1B). These results suggest that the ROS generated by the unhealthy mitochondria might play an important role in MALM.

By using the ROS scavengers *N*-acetylcysteine (NAC) and Ebselen, the role of mitochondrial ROS (mtROS) in MALM was evaluated in the irradiated HCT116 cells. Since the mtROS generation is enhanced by ionizing raddition (IR), we intended to increase the ROS generation by the mitochondria and used the IR-treated HCT116 cells. When Mieap was expressed in HCT116 cells, its signal (green) overlapped with that of the mitochondria (red), implying mitochondrial localization of Mieap (yellow; Figure 1C). However, treatment with NAC and Ebselen fully inhibited mitochondrial localization of Mieap, despite the high level of expression and the IR treatment (Figure 1C). Additionally, in the absence of ROS scavengers, the mitochondrial signal (red) merged with that of lysosomes (green) due to enforced expression of Mieap in HCT116 cells, suggesting that MALM was induced by Mieap (Figure 1D). However, as in the parental HCT116 cells, MALM was completely blocked by NAC or Ebselen (Figure 1D). These results suggest that Mieap recognizes and targets the ROS-generating mitochondria as damaged and unhealthy organelles. Further, the mtROS level generated by mitochondria plays an important role in MALM in order to eliminate the damaged and oxidized proteins in unhealthy mitochondria.

### NIX is an essential mediator for MALM

**Figure 2:**
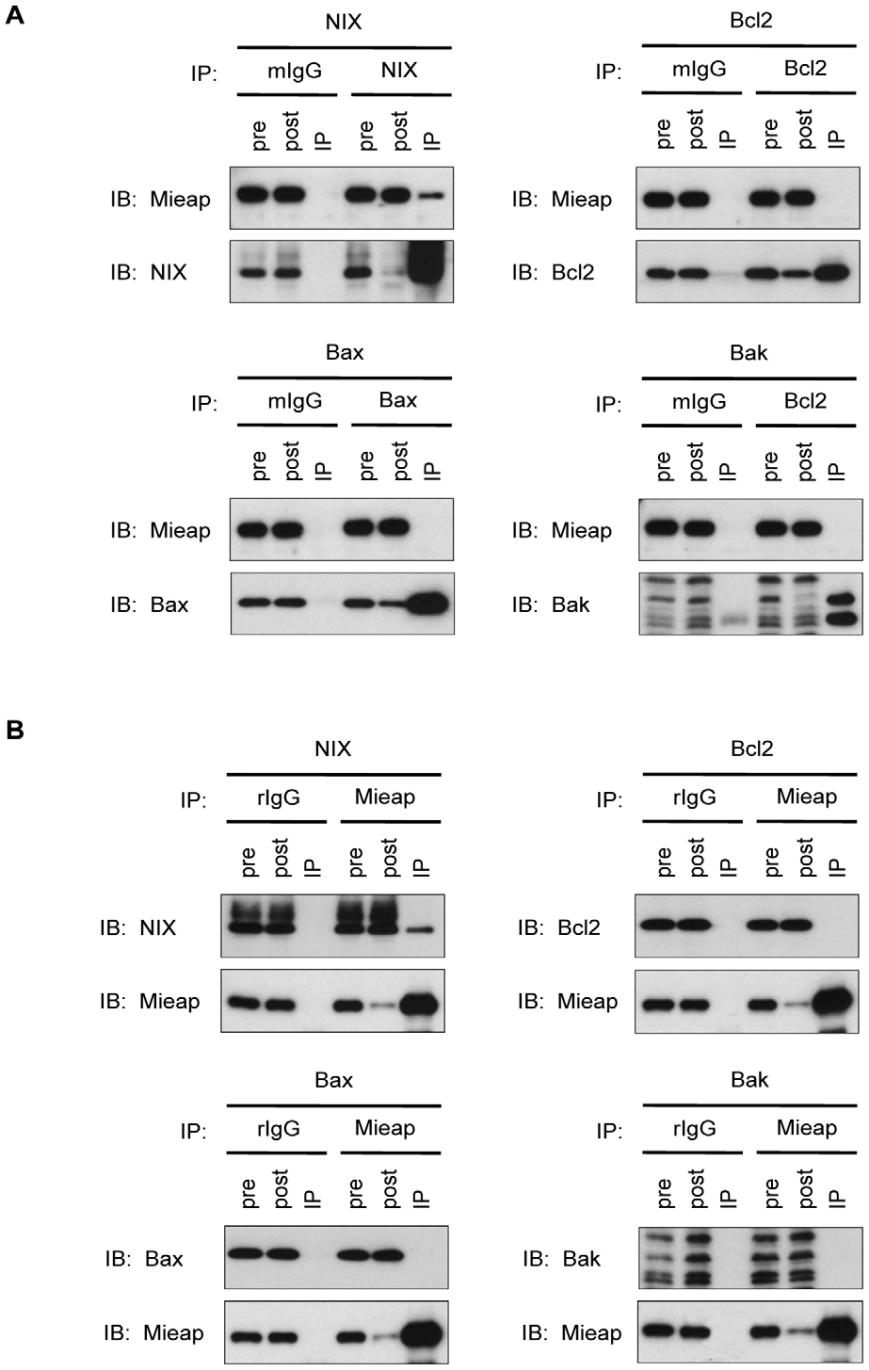
Mieap interacts with NIX, but not Bcl-2, Bax, and Bak. (A), (B) Immunoprecipitation experiment. Using the plasmids designed to express NIX, Bcl-2, Bax, and Bak, IP experiments were carried out to examine the interaction between Mieap and NIX, Bcl-2, Bax, or Bak proteins. HCT116 cells were transfected by the plasmid designed to express the N-FLAG-tagged NIX, Bcl2, Bax, or Bak, and 2 h after the transfection, the cells were infected with adenovirus vector designed to express Mieap at an MOI of 5. 36 h after the infection, the cell lysates were subjected to IP experiment as described in Materials and Methods. IP: immunoprecipitaion, IB: immunoblotting, pre: cell lysate from HCT116 cells before immunoprecipitaion, post: cell lysate from HCT116 cells after immunoprecipitation, mIgG: mouse IgG, rIgG: rabbit IgG.

To clarify the underlying mechanisms involved in MALM, we attempted to identify the Mieap-binding partner(s). Under normal conditions, Mieap is a cytoplasmic protein, but during cellular stresses (e.g., IR, ADR, or H_2_O_2_), Mieap is rapidly localized to the mitochondrial outer membrane and induces MALM. Therefore, we hypothesized that Mieap may interact with mitochondrial outer-membrane proteins to target and enter the ROS-generating mitochondria. We tested whether Mieap interacts with several mitochondrial outer-membrane proteins, including Bcl-2, Bax, Bak, and NIX. Of these proteins, we found that only NIX could be co-immunoprecipitated with Mieap (Figure 2).

**Figure 3:**
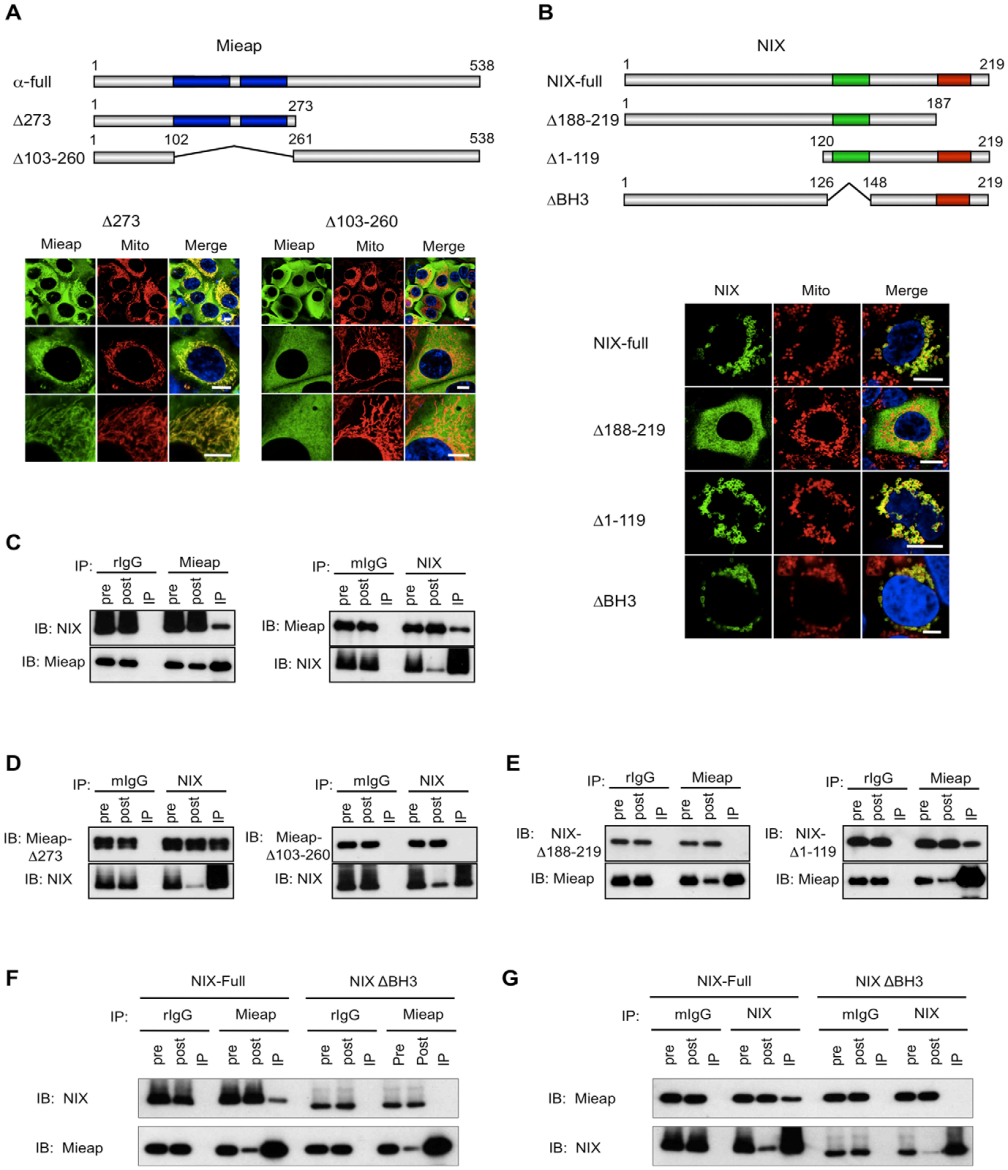
NIX interacts with the coiled-coil regions of Mieap via its BH3 domain. (A) Deletion mutants of Mieap. The expression vectors for two deletion mutants (Δ273 and Δ103-260) of Mieap were prepared, as well as Ad-Mieap-α ~full. IF experiment was carried out with anti-Mieap antibody (Mieap: green) and DsRed-mito (Mito: red). HCT116 cells were infected with Ad-Mieap vectors at an MOI of 5. Scale bar  = 10 µm. (B) Deletion mutants of NIX. The expression vectors for NIX-full and three deletion mutants (NIXΔ188-219, NIXΔ1-119, and NIXΔBH3) of NIX were prepared (upper). The subcellular localization of NIX-full and NIX mutants was shown (lower). Red box indicates transmembrane region. Green box indicates BH3 region. IF experiment was carried out with anti-FLAG antibody (NIX: green) and DsRed-mito (Mito: red). HCT116 cells were transfected by the plasmids designated to express NIX and NIX mutants. Scale bar  = 10 µm. (C–G) NIX interacts with Mieap via the coiled-coil domains of Mieap and the BH3 domain of NIX. Immunoprecipitation experiment with various forms of Mieap and NIX was carried out to characterize the interaction of Mieap and NIX (C, E, F, and G). IP: immunoprecipitaion, IB: immunoblotting, pre: cell lysate from HCT116 cells before immunoprecipitaion, post: cell lysate from HCT116 cells after immunoprecipitation, mIgG: mouse IgG, rIgG: rabbit IgG.

To characterize the interaction between Mieap and NIX, two Mieap mutants and two NIX mutants were generated (Figure 3A and 3B). While both Mieap mutants were unable to induce MALM (Figure S1), the MieapΔ273 mutant clearly localized to, and entered, the mitochondria without lysosomal accumulation, whereas the MieapΔ103–260 did not (Figure 3A). These findings suggest that the coiled-coil motif of Mieap plays a pivotal role in the targeting of and entry to the damaged mitochondria. As reported previously, NIXΔ1–119 and NIX localized to the mitochondrial outer membrane, but NIXΔ188–219 did not (Figure 3B) [9].

We next carried out co-immunoprecipitation experiments with the mutant Mieap and NIX proteins. MieapΔ273 and Mieap-full interacted with NIX, but MieapΔ103–260 was unable to bind to NIX, suggesting that Mieap interacts with NIX via the coiled-coil domain (Figure 3C–D). Furthermore, NIXΔ1–119, but not NIXΔ188–219, was able to bind to Mieap-full suggesting that the mitochondrial localization of NIX and, most likely, its BH3 domain are critical components of this interaction (Figure 3E). We also confirmed that Mieap is colocalized with NIX at mitochondrial outer membrane (Figure S2).

We further prepared the NIX mutant, which does not contain the BH3 domain (designated NIXΔBH3), in order to examine the role of the BH3 domain in the interaction of NIX with Mieap. As expected, NIXΔBH3 was able to localize to mitochondria (Figure 3B), but failed to interact with Mieap-full (Figure 3F and 3G). Taken together, these results suggest that Mieap interacts with NIX at the mitochondrial outer membrane via the coiled-coil region of Mieap and the BH3 domain of NIX.

**Figure 4:**
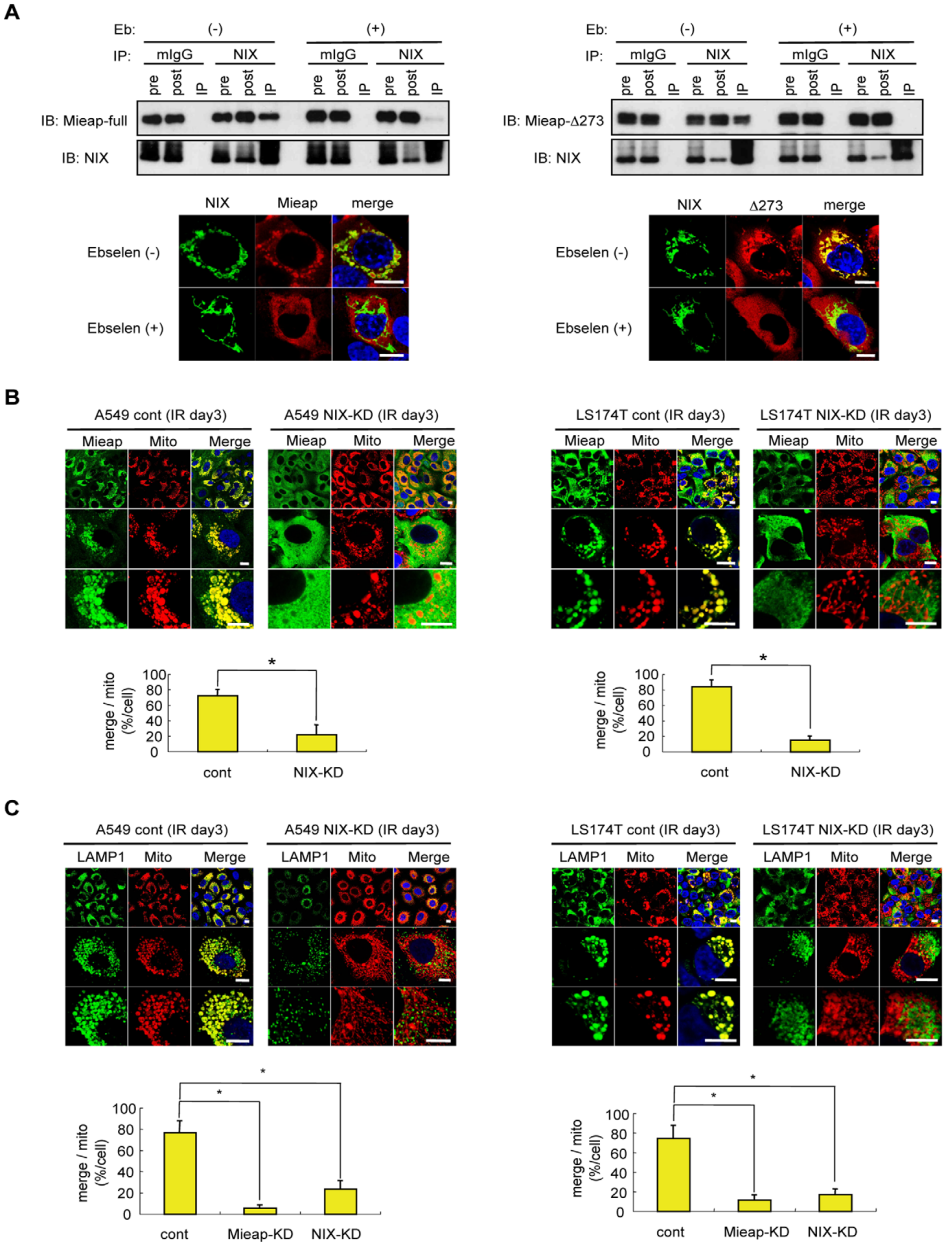
NIX is an indispensable mediator for MALM. (A) ROS plays a critical role in the interaction of Mieap and NIX. The IP (upper) and IF (lower) experiments for Mieap and NIX or for MieapΔ273 and NIX were carried out in the presence (+) or absence (−) of Ebselen. IP: immunoprecipitaion, IB: immunoblotting, pre: cell lysate from HCT116 cells before immunoprecipitaion, post: cell lysate from HCT116 cells after immunoprecipitation, mIgG: mouse IgG Scale bar  = 10 µm. (B) NIX deficiency inhibits mitochondrial localization and accumulation of Mieap. A549- or LA174T-cont and NIX-KD cells were γ-ray-irradiated, and 3 days after IR, the cells were subjected to IF analysis. Mieap protein was stained with anti-Mieap antibody (Mieap: green). Mitochondria were indicated by the DsRed-mito protein signal (Mito: red). The yellow area indicated mitochondrial localization and accumulation of Mieap. Quantitative analysis of the yellow or red area was carried out in 300–400 cells. Average values for the ratio of yellow to red (merged/mitochondrial; yellow bar graph) are shown with error bars indicating 1 SD. p<0.01 (*) was considered statistically significant. Scale bar  = 10 µm. (C) NIX deficiency inhibits MALM. Using the protocol described in (B), A549- or LS174T-NIX-KD and Mieap-KD cells were also analysed on MALM. Lysosomes were stained with anti-LAMP1 antibody (LAMP1: green), and mitochondria were indicated by the DsRed-mito protein signal (Mito: red). The yellow area indicated accumulation of lysosomes into mitochondria. Quantitative analysis of the yellow or red area was carried out by the procedure described in panel (B). The average values of the ratio of yellow to red (merged/mitochondrial; yellow bar graph) are shown with error bars indicating 1 SD. p<0.01 (*) was considered statistically significant between the control cells and Mieap-KD cells or NIX-KD cells. Scale bar  = 10 µm.

Since ROS plays a pivotal role for MALM in targeting mitochondria, we speculated that ROS might affect the interaction of Mieap with NIX. By using ROS scavengers, we examined the effects of ROS on this interaction. The treatment with Ebselen substantially inhibited the interaction of NIX with Mieap-full or MieapΔ273 (Figure 4A). Consistently, in the absence of ROS, both Mieap-full and MieapΔ273 were not co-localized with NIX at the mitochondrial outermembrane (Figure 4A). These findings indicate that the ROS generated by mitochondria may play a critical role in the interaction of Mieap and NIX, leading to the accumulation of lysosomes into mitochondria.

To evaluate the functional role of NIX in MALM, we prepared NIX-knockdown cells using A549 and LS174T (A549–NIX-KD and LS174T–NIX-KD, respectively) (Figure S3). Interestingly, the mitochondrial localization and accumulation of Mieap following IR was significantly impaired in these NIX-deficient cells (Figure 4B). Accordingly, IR-induced MALM was also dramatically inhibited in both the A549 and the LS174T NIX-KD cells (Figure 4C). These results clearly suggest that NIX is an essential factor for Mieap to target the unhealthy mitochondria.

### Mieap induces vacuole-like structures that engulf and degrade unhealthy mitochondria

**Figure 5:**
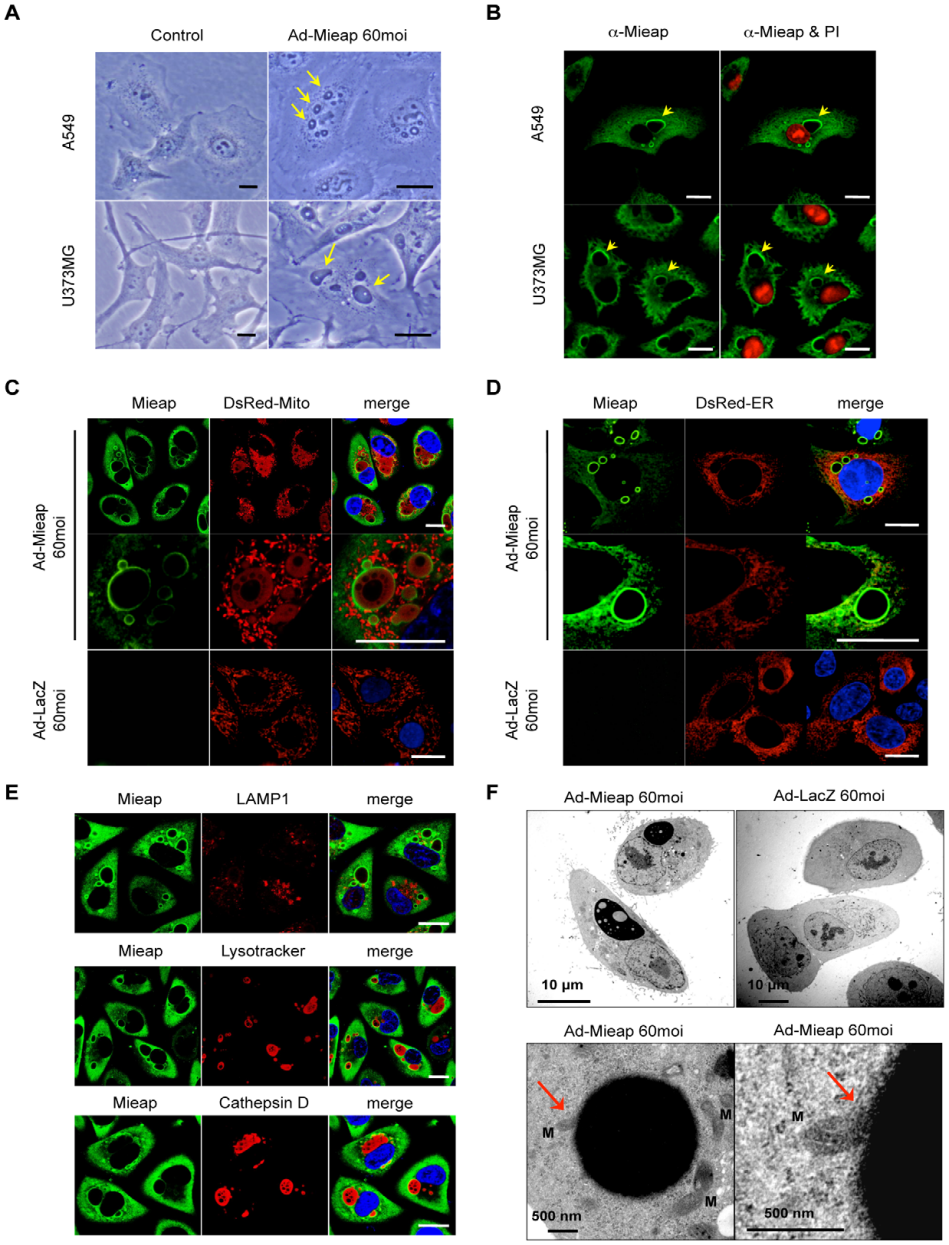
Mieap induces vacuole-like structures in order to eliminate unhealthy mitochondria. (A) Light microscopic analysis of MIV. MIVs are detected by light microscopy (LM) in A549 and U373MG cells infected with Ad-Mieap at an MOI of 60. Arrows indicate MIVs. Scale bar  = 20 µm. (B) IF analysis of MIV. Mieap (green) signal is detected around the edge of MIV. Arrows indicate MIVs. Scale bar  = 20 µm. (C) MIV eats mitochondria. Mitochondria indicated by DsRed-mito are engulfed by MIV in A549 cells infected with Ad-Mieap at an MOI of 60. Mitochondria in A549 infected with Ad-LacZ at an MOI of 60 are shown as a negative control. Scale bar  = 20 µm. (D) MIV does not eat endoplasmic reticulum. Endoplasmic reticulum (ER) indicated by DsRed-ER is not engulfed by MIV in A549 cells infected with Ad-Mieap at an MOI of 60. ER in A549 infected with Ad-LacZ at an MOI of 60 is shown as a negative control. Scale bar  = 20 µm. (E) MIV contains active lysosomal enzymes. IF analysis of A549 infected with Ad-Mieap at an MOI of 60 was carried out with anti-Mieap antibody (green) and anti-LAMP antibody (red), anti-cathepsin D antibody (red), or Lysotracker-red (red). The strong red signals of cathepsin D and Lysotracker-red were detected within MIV. Scale bar  = 20 µm. (F) Electron microscopy. MIV was examined by electron microscopy (EM). Black and large vacuole-like structures are detected in A549 infected with Ad-Mieap at an MOI of 60, which contain various sizes of vesicles. MIV directly engulfes mitochondria (indicated by arrows). Mitochondria are indicated by “M”. A549 cells infected with Ad-LacZ at an MOI of 60 are shown as a negative control. Scale bar  = 500 nm or 10 µm.

During our studies examining the function of Mieap, we noticed another unique phenomenon induced by Mieap. When Mieap protein was overexpressed in cancer cells by infection with Ad-Mieap at an MOI of 60, a few large, vacuole-like structures appeared in the cytoplasm of the infected cells (Figure 5A). The phenomenon was confirmed in all the examined cancer cell lines. We designated this vacuole-like structure as MIV (Mieap-induced vacuole). Interestingly, Mieap was localized around the edge of the MIV (Figure 5B).

In order to characterize the MIV, we first examined the relationship between the MIV and mitochondria. The cells infected with Ad-Mieap at an MOI of 60 were co-stained with an anti-Mieap antibody (green) and DsRed-mito (red). Surprisingly, the mitochondrial signal was detected within the MIV (Figure 5C). A similar result was seen using AcGFP-mito (Figure S4). We further examined the relationships between the MIV and the ER and between the MIV and Golgi. However, neither the ER signal (Figure 5D) nor the Golgi signal (Figure S5) was detected within the MIV. This result suggests that the MIV specifically targets the mitochondria.

We next examined the relationship between the MIV and the lysosomes. Lysosomes were stained with an anti-LAMP1 antibody (red), and the signal was compared to the Mieap signal (green). As shown in Figure 5E and Figure S6, the LAMP1 signal accumulated around the edge of the MIV. We also examined the cathepsin D signal and detected a strong signal inside the MIV (Figure 5E and Figure S6). We further examined the acidic status inside the MIV using Lysotracker-red. Interestingly, the inside of the MIV was strongly stained by Lysotracker-red, suggesting that it is acidic and that the lysosomal enzymes in the MIV are active (Figure 5E and Figure S6). These observations suggest that lysosomes accumulate around the edge of a MIV and fuse to the MIV membrane, after which the lysosomal contents, including digestive enzymes, are released into the MIV, leading to the degradation of the mitochondrion. Therefore, we conclude that the MIV specifically engulfs and degrades mitochondria.

To validate the role of the MIV in mitochondrial degradation, we used flow cytometry after staining the cells with Rhodamine123 (mitochondria) and NBD-C6-ceramide (Golgi). The mitochondrial staining intensity in HCT116 after infection with Ad-Mieap at an MOI of 60 was dramatically decreased in a time-dependent manner (Figure S7). In addition, the intensity of the Golgi staining was not affected by infection with Ad-Mieap, like Ad-LacZ (Figure S7). The results clearly indicate that the MIV specifically engulfs, degrades and eliminates mitochondria.

To further explore the nature of the MIV and the mechanism for how MIVs engulf mitochondria, we performed electron microscopy. The MIVs appeared as an osmium-positive, large, vacuole-like structure (Figure 5F and Figure S8). The MIVs contained a number of clear vesicles of various sizes (small to large) and directly engulfed mitochondria (Figure 5F and Figures S8). Some picture indicated that MIV-mediated uptake of mitochondria is similar to yeast microautophay (Figure S8).

We further carried out the IF experiment to examine a relationship between a mitochondrial outermembrane protein NIX and MIV. Consistent with the result in electron microscopy, NIX was degraded within MIV as soon as MIV engulfed mitochondria (Figure S9), implying that mitochondria are not likely to fuse to MIV, but rather be directly engulfed by MIV in a specific manner that may be similar to selective microautophagy in yeast (24).

**Figure 6:**
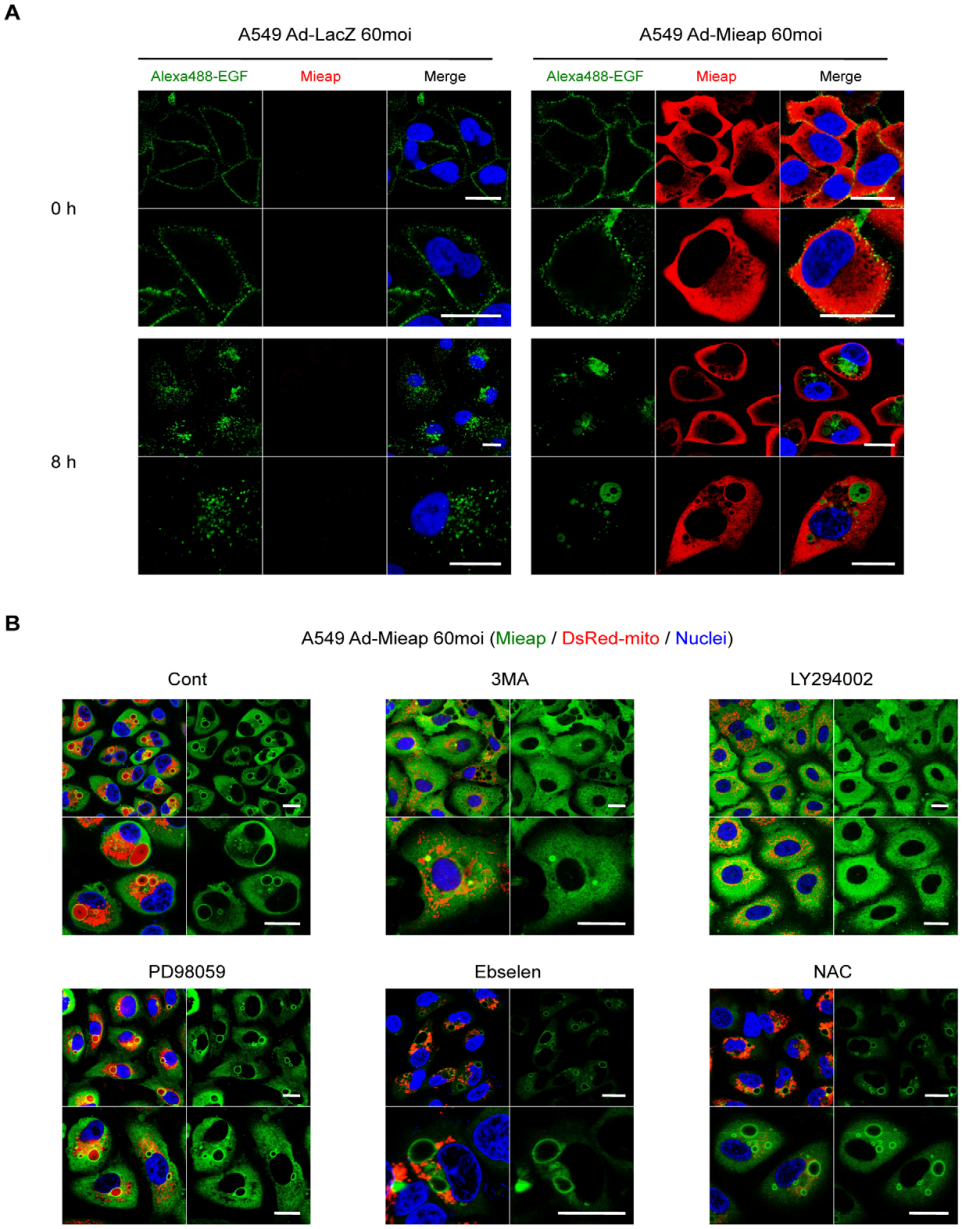
MIV generation is related to the endocytic pathway. (A) EGF incorporated into the cell by endocytosis accumulates within the MIV. Alexa488-EGF was added to the culture medium of Ad-Mieap-infected A549 cells before MIVs were generated (20 h after infection). At 0 or 8 h after the addition, the IF experiment was carried out. Mieap protein was stained with polyclonal rabbit anti-Mieap antibody (red). The added EGF was indicated by the Alexa488-EGF signal (green). Scale bar  = 20 µm. (B) MIV generation is inhibited by PI3K inhibitors. The indicated inhibitors were added to the culture medium of Ad-Mieap-infected A549 cells 6 h after infection; 36 h after infection, IF experiments were carried out. Mieap protein was stained with polyclonal rabbit anti-Mieap antibody (green). Mitochondria were indicated by the DsRed-mito protein signal (red). Scale bar  = 20 µm.

In order to characterize the nature of the MIVs, we carried out additional experiments. Since the MIVs are large vacuoles that require a large amount of membrane components, we hypothesized that the membranes of the MIVs may be supplied by the endocytotic pathway. In order to evaluate this hypothesis, we examined the relationship between endocytosis and the MIVs by carrying out an IF experiment using Alexa488-EGF. As shown in Figure 6A, a large portion of the labeled EGF accumulated within the MIVs by 8 h after the addition, suggesting that MIV generation is strongly related to the endocytotic pathway.

We further examined the effect of inhibitors against various signaling pathways on MIV generation. Interestingly, two inhibitors of phosphoinositide 3-kinase (PI3K), 3-methyladenine (3MA) and LY294002, severely impaired MIV generation, whereas ROS scavengers, Ebselen and NAC, inhibited the uptake of mitochondria by the MIVs (Figure 6B) under a condition where the Mieap protein was overexpressed. We also confirmed that Ebselen and NAC inhibited MIV generation in the irradiated NIX-KD cells (data not shown), implying that mtROS may also be required for MIV formation under physiological expression levels of Mieap after cellular stresses.

In yeast, lipid oxidation of mitochondrial outer membrane was suggested to be critical in mitochondrial autophagy (26). Therefore, we evaluated the effect of resveratrol on MALM and MIV. As shown in Figure S10, resvetatrol almost completely inhibited MALM and uptake of unhealthy mitochondria by MIV, just like Ebselen. We could not find any difference between resveratrol and Ebselen.

### The p53–Mieap pathway controls mitochondrial quality by repairing or eliminating unhealthy mitochondria via MALM or MIV

**Figure 7:**
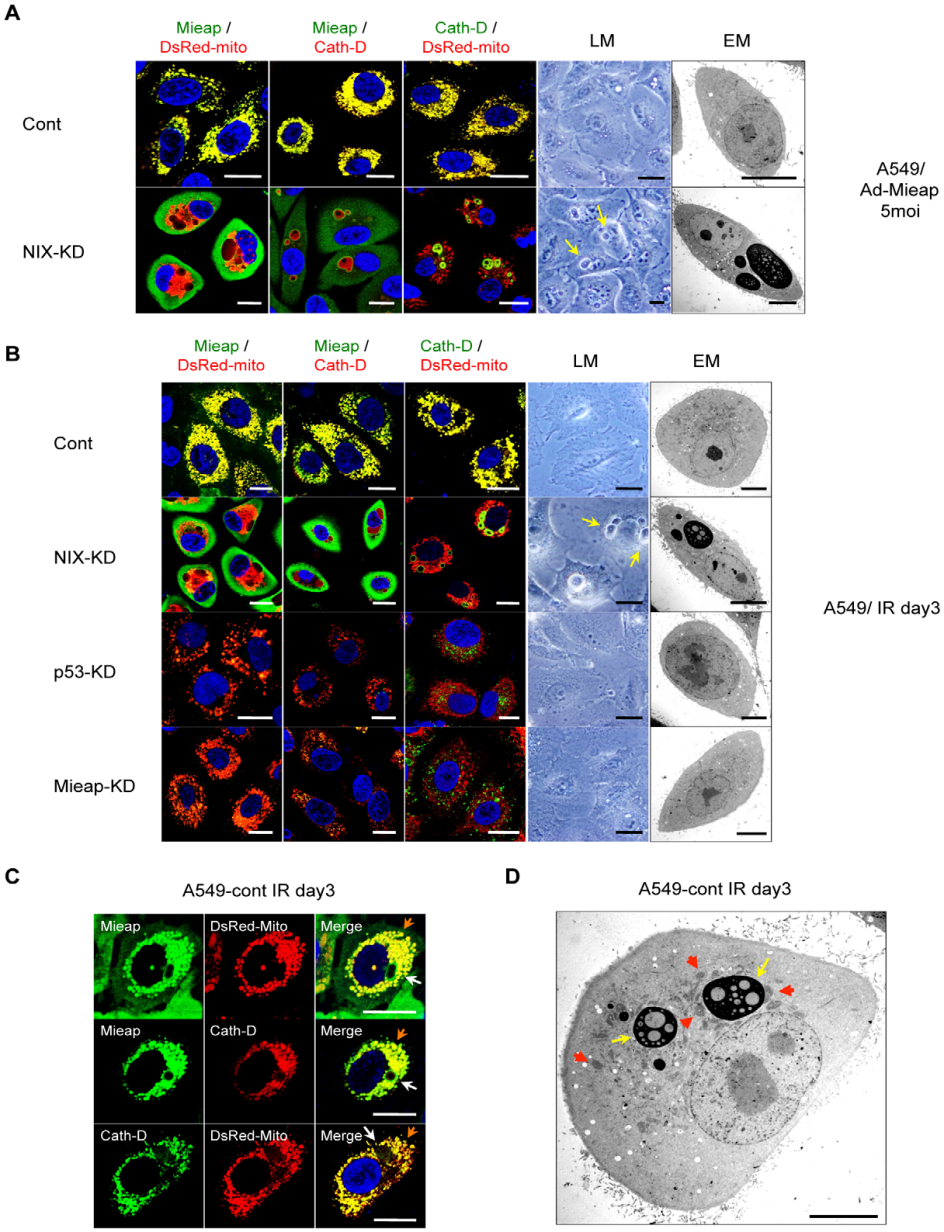
The p53-Mieap pathway repairs or eliminates unhealthy mitochondria. (A) Mieap induces MIV as an alternative pathway. A549-cont and NIX-KD cells were infected with Ad-Mieap at an MOI of 5, and 3 days after infection, IF experiment was carried out with anti-Mieap antibody (Mieap: green), or anti-cathepsin D antibody (Cath-D: green or red). Mitochondria were stained by DsRed-mito protein (DsRed-mito: red). Light microscopy (LM) and electrom microscopy (EM) experiments were also carried out with the same cells. Arrows indicate MIVs. Scale bar  = 20 µm (IF and LM) or 10 µm (EM). (B) Endogenous p53 and Mieap regulate both MALM and MIV. A549-cont, NIX-KD, p53-KD and Mieap-KD cells were irradiated by γ ray, and 3 days after IR, the experiments of IF, LM, and EM were carried out by the procedure described in panel (A). Arrows indicate MIVs. Scale bar  = 20 µm (IF and LM) or 10 µm (EM). (C) MALM and MIV are the physiological functions induced by p53. A549-cont cells were irradiated by γ ray, and 3 days after IR, the IF experiment was carried out. Mieap (green) and cathepsin D (green or red) were stained with anti-Mieap antibody and anti-cathepsin antibody, respectively. Mitochondria (red) were indicated by DsRed-mito. Orange arrows indicate MALM, and yellow arrows indicate MIV. Scale bar  = 20 µm. (D) Electron microscopy. The same cells in panel (C) were examined by electron microscopy. Red arrows indicate MALM, and white arrows indicate MIV. Scale bar  = 10 µm.

MALM plays a role in repairing mitochondria, whereas MIVs degrade mitochondria. Therefore, we hypothesized that if MALM is inhibited, MIVs should be enhanced in order to eliminate the unhealthy mitochondria as an alternative pathway for mitochondrial quality control. To evaluate this hypothesis, we examined the status of the MIVs in NIX-KD cells, in which MALM is severely impaired (see Figure 4B and 4C). Infection with Ad-Mieap at an MOI of 5 induced MALM in almost all A549-control cells (Figure 7A). However, in A549–NIX-KD cells, infection with Ad-Mieap was not able to induce MALM but dramatically induced only MIVs (Figure 7A).

To validate these results under more physiological conditions, we analyzed MALM and MIVs in IR-treated A549-control and NIX-KD cells. When the mitochondria were damaged by IR, endogenous Mieap induced MALM in all the control cells (Figure 7B). In contrast, endogenous Mieap failed to induce MALM but dramatically induced MIVs in the IR-treated NIX-KD cells (Figure 7B). Furthermore, both MALM and MIV generation were severely impaired in the IR-treated Mieap-KD cells (Figure 7B).

**Figure 8:**
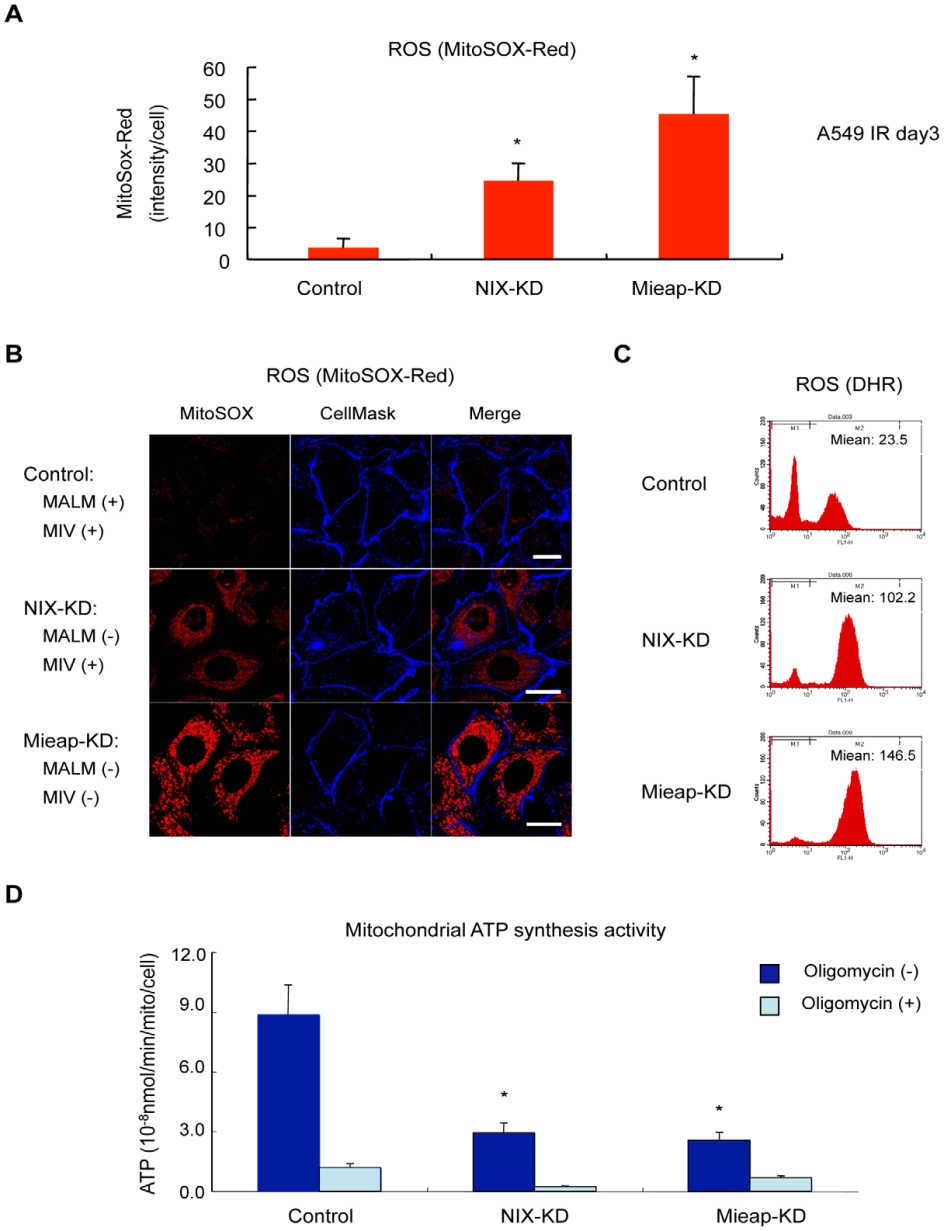
MIV eliminates unhealthy mitochondria. (A) (B) (C) Mitochondrial ROS level. The control, NIX-KD, and Mieap-KD cells of A549 were γ-ray-irradiated, and the ROS generated by mitochondria in the cells were analyzed by MitoSox-Red on day 3 after IR. (A) Quantitative analysis of ROS was carried out in 300–400 cells (lower panel). Average intensities of ROS per cell are shown with error bars indicating 1 SD. p<0.01 (*) was considered statistically significant. (B) The representative images are shown. Scale bar  = 20 µm (C) The ROS level was analyzed by FACS with dihydrorhodamine (DHR). (D) ATP synthesis activity by the mitochondria. The cells were subjected to ATP synthesis assay on day 3 after IR. Oligomycin, an inihibitor of mitochondrial oxidative phosphorylation, was used in the assay in order to detect non-mitochondrial ATP synthesis activity. The average activities of ATP synthesis are shown with error bars indicating 1 SD. p<0.01 (*) was considered statistically significant.

To examine whether the MIVs actually engulf unhealthy mitochondria, we evaluated the ROS levels generated by the mitochondria and mitochondrial ATP synthesis activity in the IR-treated A549 NIX-KD cells. As shown in Figure 8A, 8B and 8C, the ROS levels in the NIX-KD cells (MALM (−), MIV (+)) were much higher than those of the control cells (MALM (+), MIV (+)), but lower than the Mieap-KD cells (MALM (−), MIV (−)). Consistent with the result on ROS, mitochondrial ATP synthesis activity in the NIX-KD cells was much lower than the control cells, but higher than the Mieap-KD cells (Figure 8D). The similar results were obtained in the LS174T cells (Figure S11). These results suggest that the MIVs may engulf unhealthy mitochondria. These results also suggest that if the damaged mitochondria cannot be repaired by MALM, MIVs function as an alternative pathway in mitochondrial quality control by eliminating the unhealthy mitochondria. Therefore, Mieap regulates mitochondrial quality control by repairing or eliminating unhealthy mitochondria through MALM or MIVs, respectively.

Initially, we identified *Mieap* as a p53-inducible gene. Therefore, we reasoned that p53 might be involved in mitochondrial quality control. We examined whether p53 regulates MALM and the generation of MIVs in IR-treated A549-control and p53-KD cells. MALM was induced in the control cells but not in the Mieap-KD and NIX-KD cells (Figures 7B). Interestingly, both MALM and MIVs were observed in a fraction of the control cells (5∼10%), suggesting that the MIVs are the physiological function of Mieap or p53 (Figure 7C and 7D). As expected, MALM was severely inhibited in the p53-KD cells (Figure 7B). In addition, we did not observe any MIV structures in the p53-KD cells or in the Mieap-KD cells (Figure 7B). We also obtained the similar results in LS174T cells (Figure S12 and data not shown).

**Figure 9:**
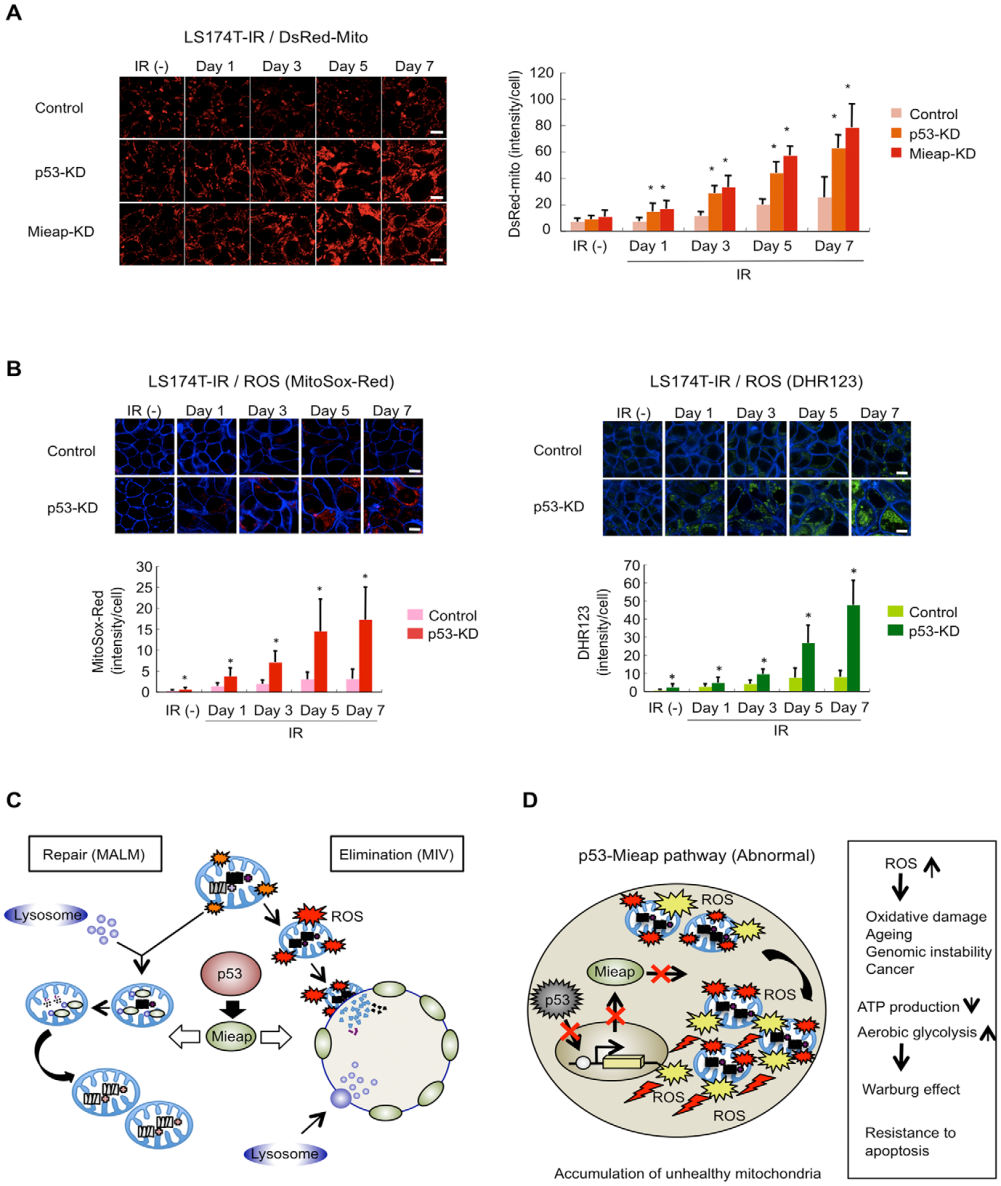
The p53-Mieap pathway controls mitochondrial quality. (A) The DsRed-mito intensity increases in p53-defective cells. The cells were γ-ray-irradiated, and the mitochondrial intensity was analyzed by the DsRed-mito signal at the indicated times. The representative images were shown (right panel). Quantitative analysis of mitochondrial intensity was carried out in 300–400 cells. Average intensities of mitochondria per cell are shown with error bars indicating 1 standard deviation (SD; left panel). p<0.01 (*) was considered statistically significant. Scale bar  = 20 µm. (B) Mitochondrial ROS level increases in p53-deficient cells. The cells were γ-ray-irradiated, and the ROS generated by mitochondria in the cells was analyzed by MitoSox-Red (red) or DHR123 (green) without IR or at the indicated times after IR. The representative images are shown (upper panel). Quantitative analysis of ROS was carried out in 300–400 cells (lower panel). Average intensities of ROS per cell are shown with error bars indicating 1 SD. p<0.01 (*) was considered statistically significant. Scale bar  = 20 µm. (C) Hypothetical model of the p53-Mieap pathway for mitochondrial quality control. (D) Hypothetical model for inactivation of the p53-Mieap pathway.

Consistent with the impairment of MALM and MIV generation, the mitochondrial volume was elevated in the p53-KD cells, as in the Mieap-KD cells (Figure 9A and Figure S13). In addition, the level of mitochondrial ROS was significantly increased after IR treatment in the p53-KD cells, implying that dysfunctional and unhealthy mitochondria accumulated in the p53-deficient cells (Figure 9B). These results suggest that p53 also controls mitochondrial quality via the Mieap pathway (Figure 9C and 9D).

## Discussion

Recently, we discovered a novel mechanism for MQC, in which Mieap, a novel p53-inducible protein, induced the intramitochondrial lysosomes that play a critical role in the elimination of oxidized mitochondrial proteins [8]. Although the ability of Mieap to recognize unhealthy mitochondria is important, the mechanism remains unclear. However, the results in the present paper may provide several clues to address this issue. First, mtROS play a key role in targeting the unhealthy mitochondria. Second, NIX, a mitochondrial outermembrane protein, is an indispensable factor in MALM; Mieap appears to localize to and recognize the unhealthy mitochondria by interacting with NIX. Third, mtROS are required for the interaction of Mieap with NIX. Therefore, we speculate that mtROS and NIX are essential factors for MALM.

The term “unhealthy mitochondria” generally refers to mitochondria with dysfunctional oxidative phosphorylation, which is manifested by reduced ATP synthesis and the excess generation of ROS [29]. Therefore, the role of mtROS in recognizing the unhealthy mitochondria seems to be very reasonable for MALM. However, since ROS are very small and soluble molecules, they are likely to diffuse from mitochondria towards the whole cytoplasm as soon as they are produced locally within mitochondria. If so, an important question is how mtROS can mark unhealthy mitochondria to be recognized by Mieap. A recent study reported that the oxidation of mitochondrial membrane lipids by mtROS might function as a tag of altered mitochondria, leading to selective autophagy of damaged mitochondria [26]. These data indicate that mtROS generated by unhealthy mitochondria oxidize and modify the mitochondrial outer membrane lipids and/or proteins that may function as a tag of unhealthy mitochondria.

In the case of MALM, we found that NIX plays a critical role in targeting unhealthy mitochondria. Modification by ROS impairs the function of some proteins, whereas other types of ROS modification reversibly modulate the function of proteins, similar to phosphorylation and dephosphorylation [30]. Therefore, we propose that mtROS generated by mitochondria modifies NIX and/or Mieap, thereby allowing NIX and Mieap to interact. Although the precise nature of the putative ROS modification of NIX and/or Mieap has not been identified, we hypothesize that the ROS-regulated modification is likely to play a crucial role in the interaction of Mieap and NIX, which allows Mieap to distinguish healthy mitochondria from unhealthy mitochondria. Further investigation on the modifications of NIX and/or Mieap by ROS is necessary.

Another important question regarding the MALM mechanism is how lysosomes enter mitochondria without destroying the structure of mitochondria. We hypothesize that an unidentified channel that is similar to the mitochondrial permeability transition pore (MPTP) may regulate lysosomal translocation from the cytoplasm to the inside of mitochondria. Interestingly, NIX was previously suggested to regulate MPTP in the context of non-apoptotic cell death [12]. Since NIX is an essential factor to induce MALM by interacting with Mieap, we propose that NIX may regulate or form a large hole that spans from the mitochondrial outer membrane to the inner membrane, leading to the translocation of lysosomes into the mitochondrial matrix under the regulation by Mieap, although further investigation is required.

In the present study, we identified an additional mechanism for Mieap-regulated MQC. Inhibition of MALM in gamma-irradiated cells induced large vacuoles in the cytoplasm in a Mieap-dependent manner. Surprisingly, the vacuoles (designated as MIVs) specifically engulfed damaged mitochondria, implying that MIV is a type of mitophagy in mammalian cells. However, electron microscopy and immunofluorescence data indicated that MIV is not related to canonical autophagy, which is mediated by double-membraned autophagosomes. In addition, the damaged mitochondria were directly contacted and engulfed by MIV. Accordingly, alternative mitophagy pathways in which the macroautophagy genes *ATG5* and *ATG12* are not essential for mitochondrial degradation have been suggested [22], [31]. We hypothesize that the machinery for the uptake and degradation of unhealthy mitochondria by MIV is likely very similar to selective microautophagy of mitochondria in yeast. Kissova et al. reported that both selective and non-selective mitophagy occur in yeast; the former was unlikely to be mediated by canonical macroautophagy or microautophagy but rather by “selective microautophagy” [24], [32]. In the selective microautophagy of mitochondria, yeast vacuoles directly contact and engulf mitochondria without engulfing the surrounding cytosol [24]. Therefore, the mechanism in yeast is likely related to the MIV-mediated mitophagy in human cells.

In yeast, the mitochondrial outer membrane protein Uth1p plays a critical role in selective microautophagy of mitochondria, but no human homolog of the protein has been identified thus far. There may be an unidentified mitochondrial outer membrane protein in human cells that plays a critical role in recognizing unhealthy mitochondria in the MIV-mediated mitophagy. NIX is unlikely to be required for targeting and degrading unhealthy mitochondria in the MIVs, since MIVs were shown to engulf and degrade mitochondria in NIX-KD cells. Interestingly, the ROS scavengers NAC and Ebselen did not inhibit the formation of MIVs, but completely inhibited the uptake of damaged mitochondria by MIVs under a condition where the Mieap protein was overexpressed. This result suggests that mtROS produced by unhealthy mitochondria might also play a critical role in targeting the MIVs to the unhealthy mitochondria. As described, the oxidation of mitochondrial outer membrane lipids was shown to be critical in selective microautophagy of mitochondria in yeast [26]. Oxidation of mitochondrial outer membrane lipids by mtROS may also function as a tag of unhealthy mitochondria for the MIV-mediated mitophagy in human cells. Alternatively, like MALM, an unidentified mitochondrial outer membrane protein and/or its modification by mtROS may play a role in targeting unhealthy mitochondria in the MIVs. Further studies are needed to understand this mechanism.

In contrast to yeast cells, there are no large vacuoles under normal conditions in the cytoplasm of mammalian cells. The MIVs were induced by gamma-ray irradiation in a p53- and/or Mieap-dependent manner. In addition, MIV generation was enhanced by inhibition of the MALM pathway. The next important question is how the MIVs are generated by Mieap. Currently, the mechanistic details remain unknown. However, the data in the present study provide several clues to address this question. First, Mieap protein was localized around the edge of vacuole-like structures, implying that Mieap itself may constitute the structure of the MIV membrane. Second, the Alexa488-EGF incorporation experiment suggested that the MIV membrane might be supplied from the cellular membrane by the endocytic pathway. Third, inhibitors of PI3K blocked MIV generation, supporting the involvement of the endocytic signaling pathway in MIV generation. On the basis of these findings, it is likely that the generation of the MIVs may be strongly related to the endocytic pathway.

We demonstrated that Mieap plays a pivotal role in mitochondrial quality control by repairing or eliminating the unhealthy mitochondria via MALM or MIVs, respectively (Figure 9C). The mechanism seems to be similar to the “p53 smart” model that regulates apoptosis [33]. In this model, p53 functions as a cell fate determinant that kills cancerous cells by inducing apoptosis or allows the cell to survive by repairing DNA damage [33]. In this context, the decision is likely to depend upon the extent of the damage. That is, when the damage is too severe to be repaired, the cell will be killed. However, if the damage is mild to moderate, the damage in the cell will be repaired and the cell will survive. Ser46-phosphorylation of p53 plays a critical role in this mechanism by selectively activating the p53 target genes involved in apoptosis [34]. Currently, the precise mechanism for the Mieap-mediated selection of repairing or eliminating the unhealthy mitochondria remains unclear. In the present study, we confirmed that the ROS level generated by the unhealthy mitochondria is essential for both MALM and MIV formation. Therefore, we speculate that the amount of ROS produced by the unhealthy mitochondria (mtROS) may play a key role in this mechanism.

Mieap primarily functions to repair the unhealthy mitochondria by inducing the MALM in order to degrade and eliminate the oxidatively damaged proteins within the mitochondria and prevent further increases in mtROS generation, thereby contributing to the healthy functioning of mitochondria (Figure 9C). However, when the damage to a mitochondrion is too severe and MALM cannot repair the damaged mitochondrion that is producing the high level of ROS, Mieap induces MIV formation in order to eliminate the severely damaged and irreparable mitochondrion (Figure 9C). Consistent with this hypothesis, the inhibition of MALM by repressing NIX dramatically induced MIV formation, and the mitochondria were engulfed and degraded by the MIVs. Therefore, MIVs play a role as an alternative and emergent system alongside MALM to maintain the healthy state of mitochondria by eliminating severely damaged mitochondria. Since MALM deficiency causes excess ROS generation by mitochondria, the increased mtROS in the cytoplasm may enhance MIV formation. We speculate that the ROS-induced modification of Mieap may be involved in the mechanism underlying the selection of MALM or MIV formation.

*p53* is an important tumor suppressor gene and is mutated in over 50% of human cancers. It encodes a sequence-specific transcription factor that activates transcription of its target genes, and we initially identified *Mieap* as a novel p53 target. Not surprisingly, *Mieap* expression was downregulated in *p53*-mutated cancers, especially following DNA damage. Hence, we reasoned that p53 might be involved in mitochondrial quality control. As expected, both MALM and MIV formation was severely impaired following IR treatment in *p53*-KD cancers, and the accumulated mitochondria generated higher levels of ROS. This clearly shows that p53 regulates mitochondrial quality control by inducing the repair or elimination of unhealthy mitochondria via MALM or MIV formation, respectively (Figure 9C). We believe p53 promotes the elimination of ROS-damaged mitochondrial proteins or promotes the degradation of damaged mitochondria via the upregulation of *Mieap*, thereby maintaining healthy organelles and preventing further increases in the ROS level. Increased ROS generation by mitochondria can cause DNA damage and genomic instability [3]; promote cell proliferation, survival and cancer metastasis [35]; and activate tumor-promoting signaling pathways such as NF-κB and AKT [36]. Therefore, preventing ROS generation by unhealthy mitochondria might be an important component of p53-mediated tumor suppression. Consistent with this hypothesis, p53 deficiency has been reported to result in increased ROS levels, and the ROS scavenger NAC can prevent lymphomas in *p53*-null mice [37], [38]. We hypothesized that one mechanism of the p53-dependent antioxidant activity might be explained by the p53–Mieap pathway (Figure 9D).

Recently, mitochondrial oxidative phosphorylation has been shown to play a critical role in Bax- and Bak-induced apoptosis [39]. Tomiyama et al. reported that ATP produced by mitochondrial oxidative phosphorylation, but not by cytoplasmic glycolysis, is required for the activation of Bax and Bak and the subsequent apoptosis. This suggests that the accumulation of dysfunctional mitochondria and impairment of mitochondrial ATP synthesis due to inactivation of the p53–Mieap pathway leads to resistance to apoptosis and contributes to cancer initiation and progression (Figure 9D). Therefore, mitochondrial quality control by the p53–Mieap pathway may also play an important role in p53-dependent apoptosis.

Although there are many important questions that require further study, the discovery of this unusual and important mechanism in the cell may lead to a greater understanding of the underlying mechanisms involved in various phenomena and diseases that may be to the result of defective mitochondrial quality control.

## Materials and Methods

### Cell lines

The following human cancer cell lines were purchased from the American Type Culture Collection: LS174T and HCT116 (colorectal adenocarcinoma); A549 (lung cancer); The TERT-immortalized normal cell lines HCK-1T (human cervical keratinocyte cell) and HMEC4 (human mammary epithelial cell) were provided by T. Kiyono (National Cancer Centre Research Institute) and D.A. Galloway (Fred Hutchinson Cancer Research Centre). Cells were cultured under the conditions recommended by their depositors.

### Immunocytochemistry

For immunocytochemistry, cells were grown on 8-well chamber slides (1×10^5^ cells/well for LS174T and 2×10^4^ cells/well for all others) at 37°C in conventional culture medium, and fixed in 2% paraformaldehyde for 10 min at room temperature. Slides were incubated with 0.1% Triton X-100 buffered in phosphate-buffered saline (PBS) for 3 min, and washed three times with PBS at room temperature. Cells were blocked with 3% bovine serum albumin (BSA) in PBS for 1 h, and sequentially incubated with rabbit polyclonal anti-Mieap antibody (1∶200) or mouse monoclonal anti-LAMP1 antibody (1∶200) for 1 h at room temperature. After washing three times with PBS, slides were incubated with fluorescein isothiocyanate (FITC)-conjugated goat anti-rabbit immunoglobulin G (IgG) antibody, FITC-conjugated goat anti-mouse IgG antibody, Alexa Fluor 546 goat anti-rabbit IgG antibody, or Alexa Fluor 546 goat anti-mouse IgG antibody for 1 h at room temperature. Slides were treated with 1 µM TO-PRO-3 (Invitrogen) for 15 min to stain the nuclei, and then washed four times with PBS. Slides were mounted with VECTASHIELD H-1000 (Vector Laboratories), and observed under an Olympus IX70 (Olympus) inverted fluorescence microscope coupled with a Radiance 2000 laser-scanning confocal system (Bio-Rad).

### Establishment of p53-KD, Mieap-KD or NIX-KD cell lines using RNA interference

We established p53-KD and Mieap-KD cell lines using LS174T and A549 cells, as described previously [8], [40]. We also established NIX-KD cell lines using LS174T and A549 cells. NIX expression was inhibited in these cell lines by retroviral expression of short-hairpin RNA (shRNA) (NIX-KD: 5′-gatccccGTTCTGTGTCTTTAAGCATttcaagagaATGCTTAAAGACACAGAACtttttggaaa-3′) against the NIX sequence. We also established control cell lines by infection with an empty retroviral vector (Control).

### Construction of recombinant adenoviruses

Replication-deficient recombinant viruses were generated and purified as described previously [41]. In brief, blunt-ended cDNAs were cloned into the SmiI site of the cosmid pAxCAwtit (Takara), which contains the CAG promoter and the entire genome of type 5 adenovirus except for E1 and E3 regions. BspT104I digested-cosmids were transfected to 293 (human embryonic kidney cell line) cells. Viruses were propagated in the 293 cells and purified.

### DNA-damaging treatments

Cells were seeded 12 h before the treatment and were 60–70% confluent at the time of the treatment. To examine the expression level of Mieap and to induce MALM, or MIV by mitochondrial damage in response to genotoxic stresses, in all the figures and supplementary figures, the cells were γ-irradiated at 60 Gy using a ^60^Co source. We confirmed that MALM and MIV were inducible by γ-irradiation at as low as 1 Gy and 10 Gy, respectively (data not shown).

### Antibodies

Rabbits were immunized with the recombinant amino (N)-terminal domain of Mieap. Antibodies were subsequently purified on antigen affinity columns. The other primary antibodies used in this study were mouse monoclonal anti-beta-actin antibody (Sigma), mouse monoclonal anti-LAMP1 antibody (BD Pharmingen), mouse monoclonal anti-FLAG antibody (Sigma), mouse monoclonal anti-cathepsin D antibody (Novus biological).

### FACS analysis

For FACS analysis of mitochondrial ROS production, the cells were incubated with 10 µM Dihydrorhodamine123 (Sigma) for 2 h at 37°C with serum-free media in the dark. Subsequently, these cells were collected by treatment with Trypsin-EDTA solution and centrifugation, washed in PBS, and re-suspended in cold PBS. Stained cells were immediately analyzed in a FACS Calibur Flow Cytometer (Becton Dickinson).

To analyze the mitochondrial intensity, the cells were incubated with 10 µM Rhodamine123 (Sigma) for 15 min at 37°C in the dark. Subsequently, the cells were isolated by centrifugation, washed in PBS and re-suspended in PBS. To analyze the Golgi, the cells were incubated with 10 µM NBD-C_6_ ceramide (Invitrogen) diluted in Hank's buffered salt solution (HBSS) for 30 min at 4°C in the dark. Subsequently, the cells were incubated with HBSS for 30 min at room temperature in the dark. Stained cells were isolated by centrifugation, washed in PBS and re-suspended in PBS. The stained cells for mitohcondria and Golgi were immediately analyzed in a FACS Calibur cytometer (Becton Dickinson).

### Quantitative analysis of accumulation of Mieap and lysosomes within mitochondria

HCT116 cells were seeded on eight-well chamber slides (1×10^5^ cells/well for LS174T and 2×10^4^ cells/well for all other cells) at 37°C in conventional media. After 12 h, the cells were infected with Ad-DsRed-Mito at an MOI of 30, and 12 h later they were infected with Ad-Mieap at an MOI of 5. 24 h after infection with Ad-Mieap, the cells were γ-ray irradiated, and 2 h after IR, the ROS scavengers, 5 mM NAC (Sigma) and 10 µM Ebselen, were added to the culture medium. 72 h after IR, the cells were subjected to IF analysis. The mitochondrial signal (red area) and the overlapping of the Mieap and mitochondrial signals (yellow area) or the overlapping of the mitochondrial and lysosomal signals (yellow area) were analyzed by LuminaVision image analysis software in 300–400 cells, as described above. Similar experiments were carried out upon the NIX-KD cells.

### Immunoprecipitation

In order to examine the interaction of Mieap with NIX, Bcl2, Bax, or Bak, HCT116 cells were transfected by the plasmid designed to express the N-FLAG-tagged NIX, Bcl2, Bax, or Bak, and 2 h after the transfection, the cells were infected with adenovirus vector designed to express Mieap at an MOI of 5. In order to confirm the interaction of Mieap with NIX, the various forms of Mieap or NIX were expressed in HCT116 by infection with adenovirus vectors designed to express Mieap and Mieap-mutants at an MOI of 5 or transfection of plasmids designed to express the N-FLAG-tagged NIX and NIX-mutants, respectively. 36 h after the infection, the cells were lysed on ice for 15 min in 500 µl NP40 lysis buffer (1% NP40, 150 mM NaCl, 25 mM Tris-HCl pH 7.6, Complete protease inhibitor cocktail (Roche)). After lysis, cell debris was removed by centrifugation at 12,000× g for 15 min, and the supernatant was collected. The supernatant was precleared by absorbing it with normal IgG and 20 µl of protein-A or Protein-G sepharose beads for 1 h at 4°C. The beads were removed by centrifugation, and the supernatant was subjected to IP by adding 1 µg rabbit polyclonal anti-Mieap antibody or 1 µg mouse monoclonal anti-FLAG antibody. As negative controls, normal rabbit IgG or normal mouse IgG was used for IP. The antibody mixtures were allowed to react on a rotating devise overnight at 4°C. Then, Protein-A or Protein-G sepharose beads were added, and the mixtures were incubated for an additional 2 h at 4°C. The beads were washed five times in cold lysis buffer, and immune complexes were released from the beads by boiling them in 2 x Laemmli sample buffer. Samples were loaded onto 10–15% SDS-PAGE gels, and electrophoresed proteins were subjected to western blot analysis with anti-FLAG antibody or anti-Mieap antibody as described above.

### Visualization of subcellular organelles

Mitochondria, ER and Golgi were visualized by infection with recombinant adenovirus vectors or transfection with plasmids designed to express organelle marker proteins such as DsRed-Mito for mitochondria, DsRed-ER for ER and AcGFP-Golgi for Golgi. Alternatively, to visualize mitochondria, cells were incubated with 50 nM MitoTracker-Red (Invitrogen) or 50 nM MitoTracker-Green (Invitrogen) for 30 min at 37°C before fixation. To visualize lysosomes, 50 nM LysoTracker-Red (Invitrogen) for 30 min at 37°C before fixation.

### Transmission electron microscopy

LS174T-control and Mieap-KD cells (1.5×10^5^ cells/24-well plate) or Ad-Mieap and Ad-LacZ infected HCT116 cells (6×10^4^ cells/24-well plate) were irradiated by γ ray at 60 Gy. 3 days after IR, the cells were fixed in 2.5% glutaraldehyde in phosphate buffered saline (PBS) at 4°C for 2 h. The cells were washed with PBS, post-fixed in 1% OsO_4_ buffered with PBS for 2 h, dehydrated in a graded series of ethanol and embedded in Epon 812. Ultrathin sections (90 nm) were collected on copper grids, double-stained with uranyl acetate and lead citrate, and then observed using transmission electron microscopy (H-7100, Hitachi).

### EGF endocytosis assay

EGF coupled to Alexa Fluor 488 (Invitrogen) was used as an endocytic probe. A549 cells were infected with Ad-Mieap and Ad-LacZ at an MOI of 60 on 8-well chamber slides. Twelve hours later after infection, cells were maintained in serum-free media for 6 h at 37°C. For EGF-uptake cells were first washed in ice-cold PBS and then incubated on ice in uptake-medium (RPMI, 2% BSA, 20 mM HEPES, pH 7.5) containing 5 µg/ml Alexa Fluor 488-EGF. After incubation on ice for 1 h, the cells were washed three times in ice-cold PBS to remove unbound ligand. Cells from one well were fixed to give the total amount of bound ligand and the remaining wells were transferred to a 37°C incubator. At each indicated time point, the cells were fixed and processed for confocal microscopy.

### Experiments with various inhibitors

A549 cells were infected with Ad-Mieap at an MOI of 60. Six hours later after infection, cells were incubated in solution containing each drug as follows: 50 µM 3-Methyladenine; 3MA, 25 µM LY294002 (Calbiochem), 25 µM PD98059 (Calbiochem), 25 mM N-acetylcysteine; NAC, 10 µM Ebselen (Sigma). 24 h after infection, the cells were fixed and processed forimmunocytochemistry.

### Quantitative analysis of ROS

ROS generation by mitochondria in living cells was analyzed with the mitochondrial superoxide indicator MitoSOX-Red (Invitrogen) or dihydrorhodamine123 (DHR123) (Sigma). The cells were seeded onto a 35-mm glass-bottomed dish (Biousing Biotechnology) (1×10^6^ cells/dish for LS174T and 2×10^5^ cells/dish for all others) at 37°C in conventional media. After 24 h, the cells were treated with or without stresses, and the ROS level was examined at the indicated times. Briefly, the cells were incubated with 5 µM MitoSOX-Red or 10 µM DHR123 for 10 min at 37°C in serum-free media, washed twice with serum-free media, and then incubated with 5 µM CellMask (Invitrogen) for 15 min to visualize the walls of living cells. After washing twice with serum free-media, the stained cells were immediately observed under the confocal laser-scanning microscope, and the images were captured using an excitation filter of 510 nm and an emission filter of 580 nm for MitoSOX-Red, and an excitation filter of 543 nm and an emission filter of 579 nm for DHR123. The intensity of MitoSOX-Red or DHR123 in each captured image was analyzed by LuminaVision image analysis software as described above.

### Assay of mitochondrial ATP synthesis

The experiment was carried out according to the procedure reported previously (8). The Control and Mieap-KD cells of LS174T (3×10^6^ cells/6 cm dish) or Ad-Mieap and Ad-LacZ infected cells of HCT116 (1×10^6^ cells/6 cm dish) were irradiated by γ ray at 30 Gy, and harvested day 7 after IR. The cells with or without the treatment were subjected to ATP synthesis assay as following. The cells (5×10^5^ cells) were collected by centrifugation at 900× g at room temperature (RT), and the cell pellet was resuspended in 160 µl of buffer A (150 mM KCl, 25 mM Tris-HCl, 2 mM EDTA, 10 mM KH_2_PO_4_, 0.1 mM MgCl_2_, 0.1% BSA, pH 7.4). 5 µl of digitonin (825 µg/ml) (Sigma) was added to the suspension (to 25 µg/ml), and incubated for 1 min at RT with gentle agitation. Digitonin was removed by washing the cells with 1 ml buffer A, and then centrifuged at 900× g at RT. The cell pellet was resuspended in 175 µl of buffer A. 5 µl of 6 mM *P^1^*, *P^5^* - di (adenosine) pentaphosphate (to 0.15 mM), 5 µl of 4 mM ADP (to 0.1 mM), 2.5 µl of 80 mM malate (to 1 mM) and 2.5 µl of 80 mM pyruvate (to 1 mM), and then 10 µl of buffer B (0.8 mM luciferin (Wako) and 20 µg/ml luciferase (Wako) in 0.5 M Tris-acetate, pH 7.75) were added to the cell suspension. Cell suspension was transferred to a luminometer cuvette. After a gentle mixing with a vortex for 2 s, the cuvette was placed in luminometer Rumat LB 9507 (Berthold technologies) and light emission was continuously recorded for 5 min (17 readings). To obtain the baseline luminescence activity corresponding to non-mitochondrial ATP production, light emission was recorded in the presence of oligomycin (to 4 µg/ml). The change in RLU (Relative light units) was then converted to ATP concentration based on an ATP standard curve. The activity was shown as ATP production per min in a cell (nmol/min/cell). The value was divided by the mean intensity of mitochondria (MitoTracker-Green) per cell in order to reflect the ATP synthesis activity of the same amount of mitochondria in a cell (nmol/min/mito/cell).

### Quantitative analysis of mitochondrial intensity

The intensity of mitochondria was monitored by DsRed-Mito. The cells were seeded on 8-well chamber slides (1×10^5^ cells/well for LS174T and 2×10^4^ cells/well for all other cells) at 37°C in conventional media, and infected with Ad-DsRed-Mito at an MOI of 30 after 12 h. At 24 h after infection, the cells were irradiated by γ ray. To examine the mitochondrial intensity at the indicated times, the cells were fixed in 2% paraformaldehyde for 20 min at room temperature (RT). The slides were then treated with 1 µM TO-PRO-3 (Invitrogen) for 20 min to stain the nuclei, and subsequently washed four times with PBS. The slides were mounted with VECTASHIELD H-1000 (Vector Laboratories), and observed under an Olympus IX70 inverted fluorescence microscope coupled with a Radiance 2000 laser-scanning confocal system (Bio-Rad). The DsRed-Mito images, each of which contained 30–40 cells, were captured using an excitation filter of 563 nm and an emission filter of 582 nm. The intensity of DsRed-Mito in each captured image was analyzed by LuminaVision image analysis software (Version 2.4). The total areas of mitochondrial intensity in 300–400 cells were extracted using the optimal threshold parameters and calculated with LuminaVision image analysis software. The mitochondrial intensity was presented as the average of the calculated values per cell with error bars. Similar experiments were carried out using anti-mitochondrial antibody AE-1 (Leinco Technologies), MitoTracker-Red (Invitrogen), and MitoTraker-Green (Invitrogen).

## Supporting Information

Figure S1 **Two deletion mutants (Δ273 and Δ103-260) of Mieap fail to induce MALM.**
The adenovirus expression vectors for two deletion mutants (Δ273 and Δ103-260) of Mieap were prepared, as well as Ad-Mieap-α ~full. HCT116 cells were infected with Ad-LacZ, Ad-Mieap-α ~full, Ad-MieapΔ273, or Ad-MieapΔ103-260, and 48 h after infection, IF experiment was carried out with anti-Mieap antibody (green or red), andi-LAMP1 antibody (green), and DsRed-mito (red). The representative images were shown. Scale bar  = 10 µm.
(TIF)

Figure S2 **NIX colocalizes with Mieap at mitochondrial outer membrane.**
HCT116 cells were infected with Ad-Mieap and Ad-NIX at an MOI of 5, and 48 h after infection, IF experiment was carried out with anti-FLAG antibody (NIX: green), and anti-Mieap antibody (Mieap: red). The representative images were shown. Scale bar  = 10 µm.
(TIF)

Figure S3 **The NIX expression is severely downregulated in the NIX-KD cells of A549 and LS174T.**
Western blot analysis indicated that the NIX expression level is severely impaired in the NIX-KD cells of A549 and LS174T, compared with the parent, control and Mieap-KD cells of A549 and LS174T. The cells were irradiated by γ ray, and on day 3 after IR, the cell lysates were subjected to western blot analysis. β-actin was used as a loading control.
(TIF)

Figure S4 **MIV eats mitochondria.**
Mitochondria indicated by AcGFP-mito were engulfed by MIV in A549 cells infected with Ad-Mieap at an MOI of 60. Mitochondria in A549 cells infected with Ad-LacZ at an MOI of 60 are shown as a negative control. IF experiment was carried out with anti-Mieap antibody (red), and AcGFP-mito (green). The representative images were shown. The white and yellow arrows indicate the MIV and the degraded mitochondria within the MIV, respectively. Scale bar  = 10 µm.
(TIF)

Figure S5 **MIV does not eat Golgi.**
Golgi indicated by AcGFP-Gologi was not engulfed by MIV in A549 cells infected with Ad-Mieap at an MOI of 60. ER in A549 infected with Ad-LacZ at an MOI of 60 is shown as a negative control. IF experiment was carried out with anti-Mieap antibody (red), and AcGFP-Golgi (green). The representative images were shown. Scale bar  = 10 µm.
(TIF)

Figure S6 **The LAMP1 signal are not detected within the MIV.**
The magnified data on Figure 5E are shown. In contrast to the Lysotracker and cathepsin D singals, we never found the LAMP1 signal within the MIV. Scale bar  = 20 µm.
(TIF)

Figure S7 **MIV specifically eats and degrades mitochondria.**
(A) (B) HCT116, LS174T, and A549 cells were infected with Ad-Mieap or Ad-LacZ at an MOI of 60. After 24 h, mitochondria or Golgi were stained by Rhodamine123 (A) or NBD-C6-ceramide (B), respectively. The signals of mitochondria or Golgi were analyzed by fluorescence activated cell sorting (FACS).
(TIF)

Figure S8 **Electron microscopic analysis on the MIVs.**
(A) Consistent with the data in other experiments (Figure 5A–E), the MIVs were detected by electron microscopic analysis as various sizes of extremely-dense and round structures. Yellow arrows indicate MIVs. N: nucleus Scale bar  = 5 µm
(B) MIV may engulf mitochondria in a manner that is similar to yeast selective microautophagy of mitochondria. Yellow arrow indicates MIV. m: mitochondria Scale bar  = 1 µm or 500 nm.
(TIF)

Figure S9 **A mitochondrial outer membrane protein NIX is degraded by MIV.**
In order to examine the relationship between NIX and MIV, the IF experiment was carried out. A549 cells were transfected by the plasmid designed to express the N-FLAG-tagged NIX, and 2 h after the transfection, the cells were infected with adenovirus vector designed to express Mieap at an MOI of 5. 36 h after the infection, the cells were subjected to IF experiment with anti-FLAG antibody (NIX: green), and anti-Mieap antibody (Mieap: red). The representative images were shown. White arrows indicate MIVs. Yellow arrows indicate the degradation of NIX within MIV. Scale bar  = 10 µm.
(TIF)

Figure S10 **Resveratrol and Ebselen inhibit MALM and uptake of mitochondria by MIV.**
To examine the effect of resveratrol and ebselen on MALM, A549 cells were infected with Ad-Mieap at an MOI of 5 and Ad-DsRed-Mito at an MOI of 30. 24 h after the infection, the cells were irradiated by γ ray, and 2 h after IR, 25 µM resveratrol or 10 µM Ebselen was added to the culture media. On day 3 after IR, the cells were subjected to IF experiment.
To examine the effect of resveratrol and ebselen on MIV, A549 cells were infected with Ad-Mieap at an MOI of 60 and Ad-DsRed-Mito at an MOI of 30. 2 h after the infection, 25 µM resveratrol or 10 µM Ebselen was added to the culture media. 24 h after the infection, the cells were subjected to IF experiment.
The IF experiment was carried out with rabbit polyclonal anti-Mieap antibody (Mieap: green), mouse monoclonal anti-LAMP1 antibody (LAMP1: green), and DsRed-Mito (mitochondria: red). Scale bar  = 20 µm.
(TIF)

Figure S11 **MIV eliminates unhealthy mitochondria.**
(A) (B) (C) Mitochondrial ROS level. The control, NIX-KD, and Mieap-KD cells of LS174T were irradiated by γ ray, and the ROS generated by mitochondria in the cells were analyzed by MitoSox-Red on day 3 after IR. (A) Quantitative analysis of ROS was carried out in 300–400 cells (lower panel). Average intensities of ROS per cell are shown with error bars indicating 1 SD. p<0.01 (*) was considered statistically significant. (B) The representative images are shown. Scale bar  = 20 µm (C) The ROS level was analyzed by FACS with dihydrorhodamine (DHR).
(D) ATP synthesis activity by the mitochondria. The cells were subjected to ATP synthesis assay on day 3 after IR. Oligomycin, an inihibitor of mitochondrial oxidative phosphorylation, was used in the assay in order to detect non-mitochondrial ATP synthesis activity. The average activities of ATP synthesis are shown with error bars indicating 1 SD. p<0.01 (*) was considered statistically significant.
(TIF)

Figure S12 **p53 controls mitochondrial quality.**
p53 regulates MALM. The cont and p53-KD cells of LS174T were subjected to IF experiment on day 3 after IR. Mieap protein was stained with polyclonal rabbit anti-Mieap antibody (Mieap: green or red). Lysosomes were stained with mouse monoclonal anti-LAMP1 antibody (LAMP1: green). Mitochondria were indicated by the DsRed-mito protein signal (Mito: red). Scale bar  = 10 µm.
(TIF)

Figure S13 **The mitochondrial intensity increases in p53-defective cells.**
The cells were irradiated by γ ray, and the mitochondrial intensity was analyzed by the signal of mouse monoclonal anti-human mitochondria antibody (Leinco Technology, MO, USA: clone AE1) at the indicated times. Quantitative analysis of mitochondrial intensity was carried out in 300–400 cells. Average intensities of mitochondria per cell are shown with error bars indicating 1 standard deviation (SD; left panel). p<0.01 (*) was considered statistically significant.
(TIF)
