# Supplementary figures and images for: Mieap, a p53-Inducible Protein, Controls Mitochondrial Quality by Repairing or Eliminating Unhealthy Mitochondria
Source: PLoS One. 2011 Jan 17;6(1):e16060. doi: 10.1371/journal.pone.0016060 (PMC3022033; doi:10.1371/journal.pone.0016060)

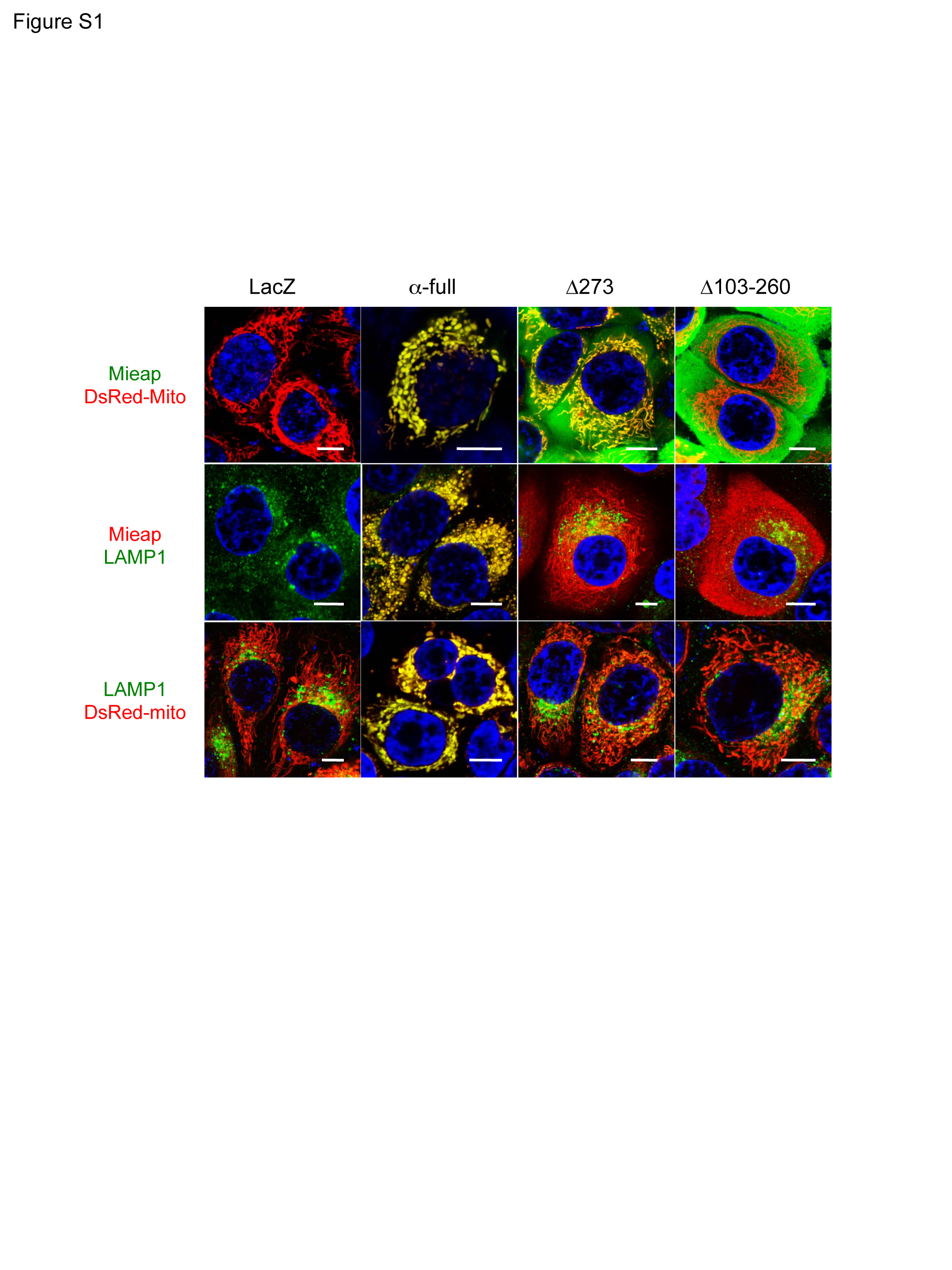

Supplement: Figure S1 — Two deletion mutants (Δ273 and Δ103-260) of Mieap fail to induce MALM. The adenovirus expression vectors for two deletion mutants (Δ273 and Δ103-260) of Mieap were prepared, as well as Ad-Mieap-α ~full. HCT116 cells were infected with Ad-LacZ, Ad-Mieap-α ~full, Ad-MieapΔ273, or Ad-MieapΔ103-260, and 48 h after infection, IF experiment was carried out with anti-Mieap antibody (green or red), andi-LAMP1 antibody (green), and DsRed-mito (red). The representative images were shown. Scale bar = 10 µm. (TIF) [file pone.0016060.s001.tif]

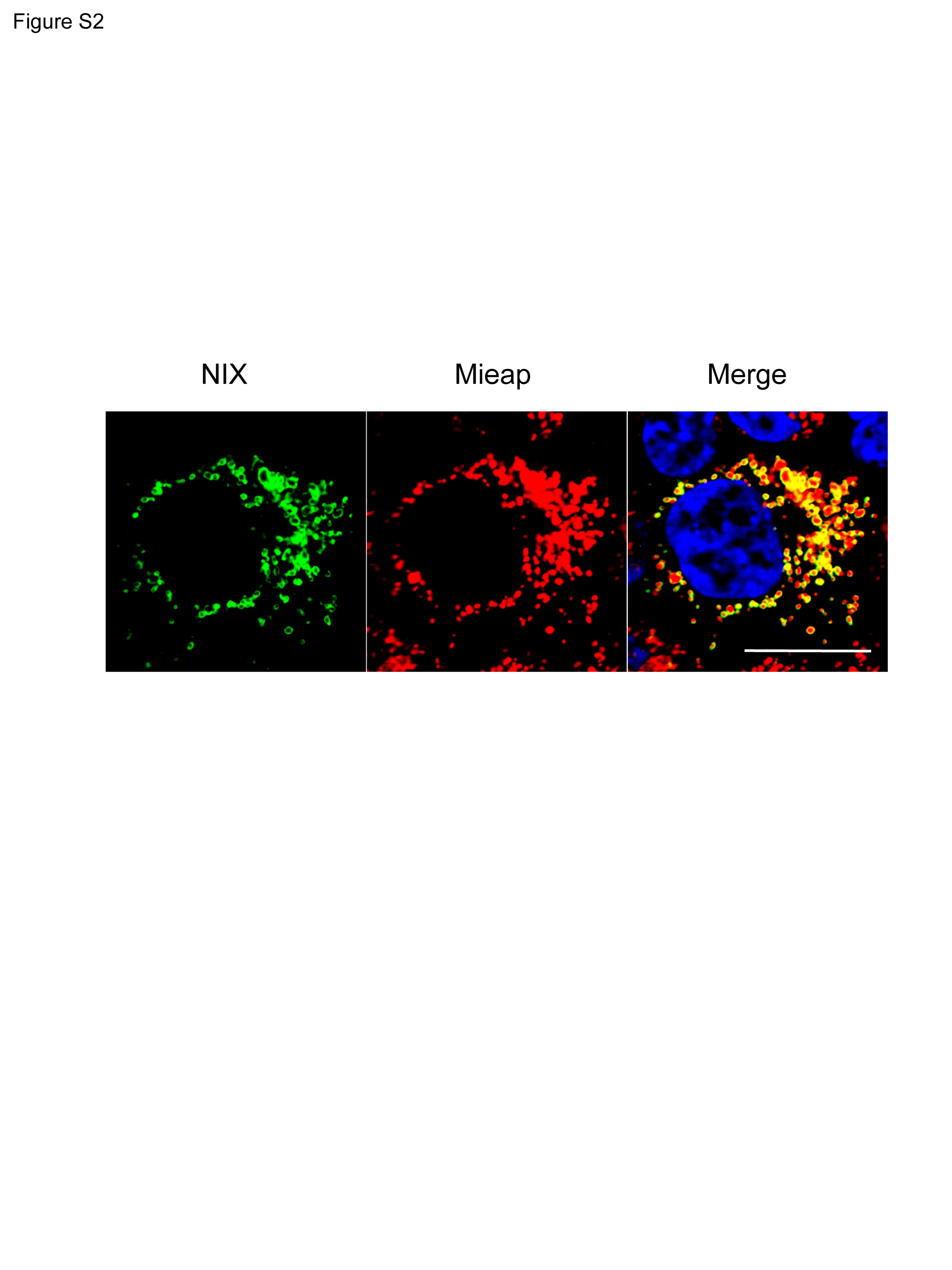

Supplement: Figure S2 — NIX colocalizes with Mieap at mitochondrial outer membrane. HCT116 cells were infected with Ad-Mieap and Ad-NIX at an MOI of 5, and 48 h after infection, IF experiment was carried out with anti-FLAG antibody (NIX: green), and anti-Mieap antibody (Mieap: red). The representative images were shown. Scale bar = 10 µm. (TIF) [file pone.0016060.s002.tif]

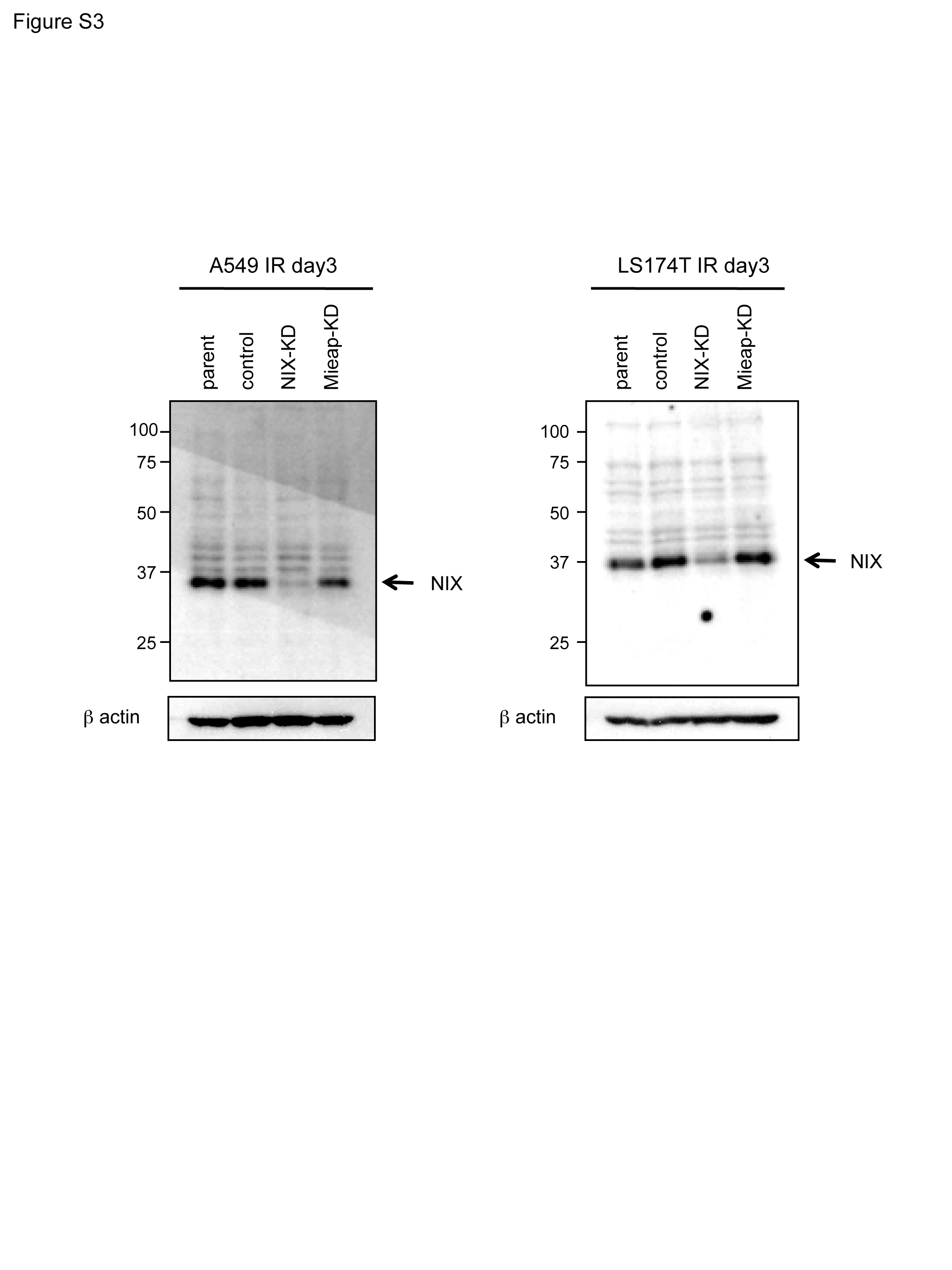

Supplement: Figure S3 — The NIX expression is severely downregulated in the NIX-KD cells of A549 and LS174T. Western blot analysis indicated that the NIX expression level is severely impaired in the NIX-KD cells of A549 and LS174T, compared with the parent, control and Mieap-KD cells of A549 and LS174T. The cells were irradiated by γ ray, and on day 3 after IR, the cell lysates were subjected to western blot analysis. β-actin was used as a loading control. (TIF) [file pone.0016060.s003.tif]

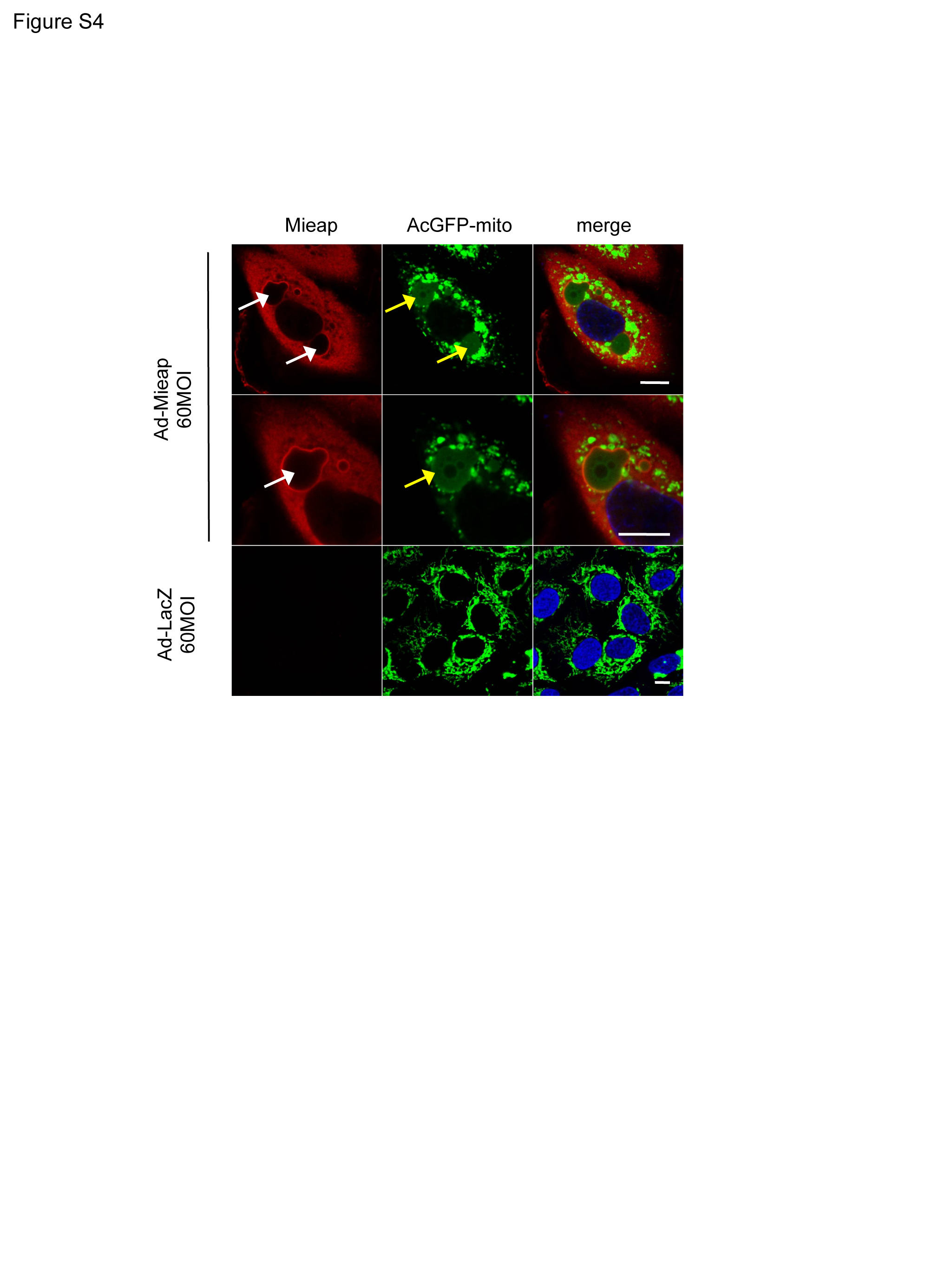

Supplement: Figure S4 — MIV eats mitochondria. Mitochondria indicated by AcGFP-mito were engulfed by MIV in A549 cells infected with Ad-Mieap at an MOI of 60. Mitochondria in A549 cells infected with Ad-LacZ at an MOI of 60 are shown as a negative control. IF experiment was carried out with anti-Mieap antibody (red), and AcGFP-mito (green). The representative images were shown. The white and yellow arrows indicate the MIV and the degraded mitochondria within the MIV, respectively. Scale bar = 10 µm. (TIF) [file pone.0016060.s004.tif]

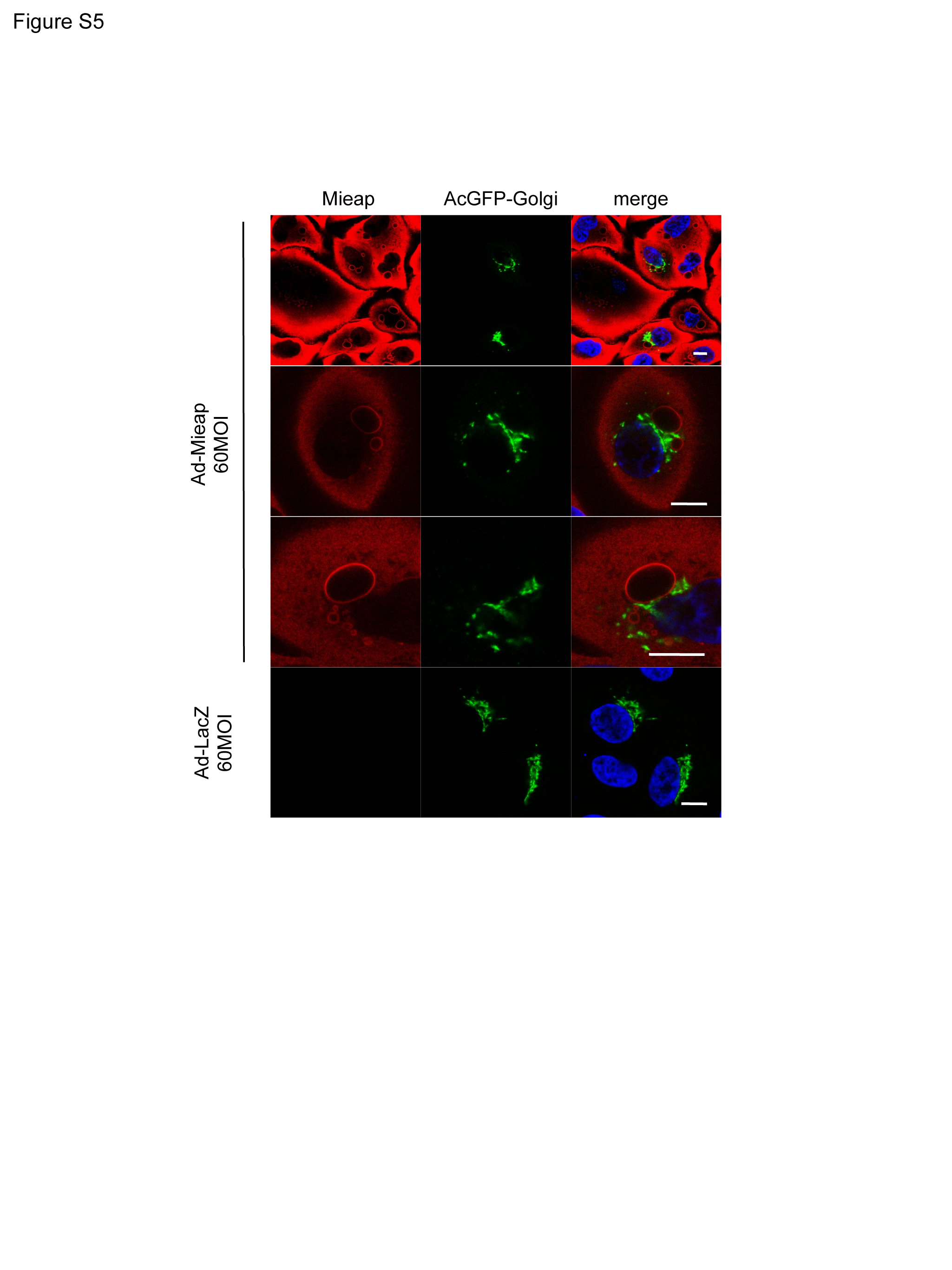

Supplement: Figure S5 — MIV does not eat Golgi. Golgi indicated by AcGFP-Gologi was not engulfed by MIV in A549 cells infected with Ad-Mieap at an MOI of 60. ER in A549 infected with Ad-LacZ at an MOI of 60 is shown as a negative control. IF experiment was carried out with anti-Mieap antibody (red), and AcGFP-Golgi (green). The representative images were shown. Scale bar = 10 µm. (TIF) [file pone.0016060.s005.tif]

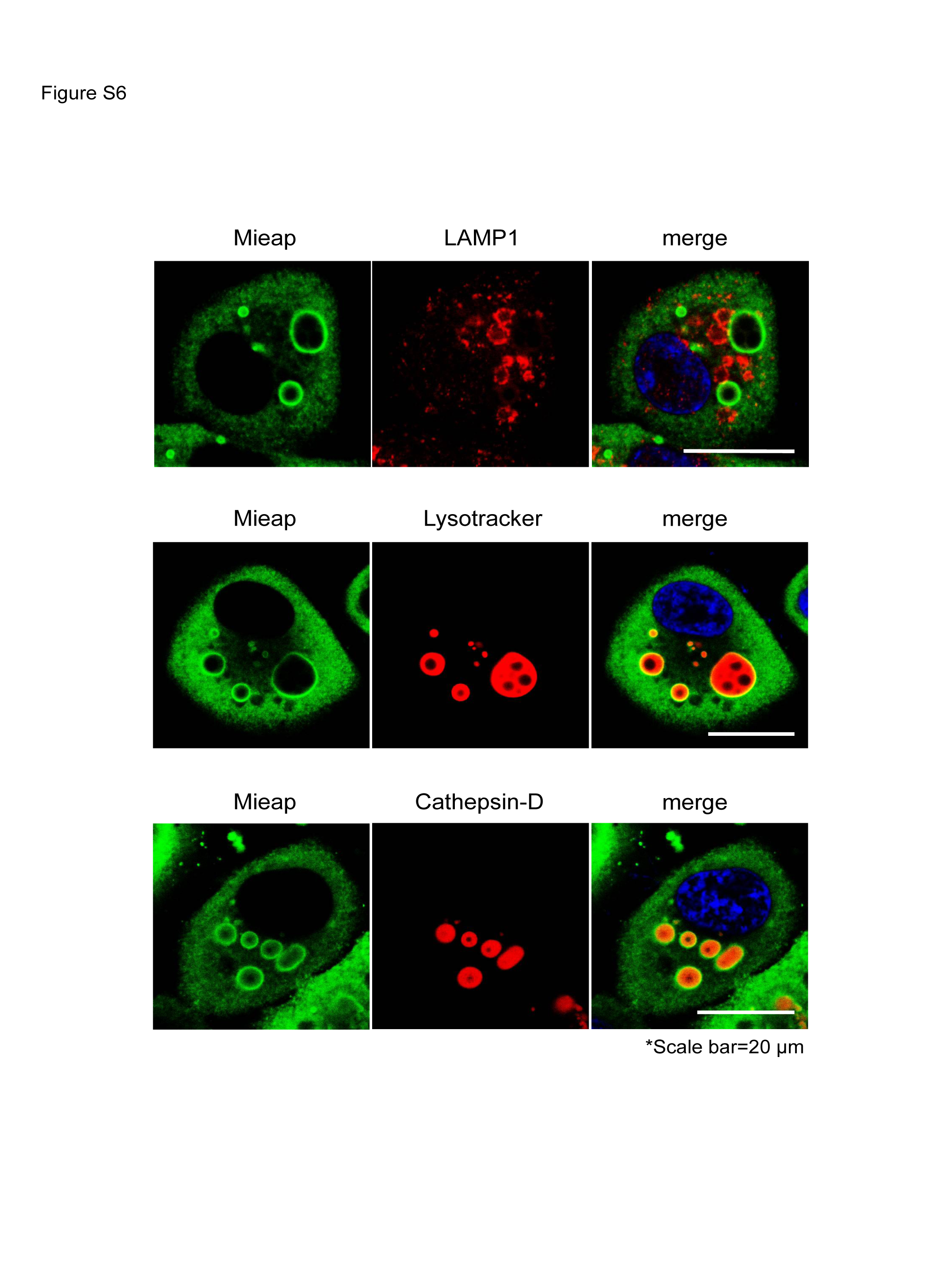

Supplement: Figure S6 — The LAMP1 signal are not detected within the MIV. The magnified data on Figure 5E are shown. In contrast to the Lysotracker and cathepsin D singals, we never found the LAMP1 signal within the MIV. Scale bar = 20 µm. (TIF) [file pone.0016060.s006.tif]

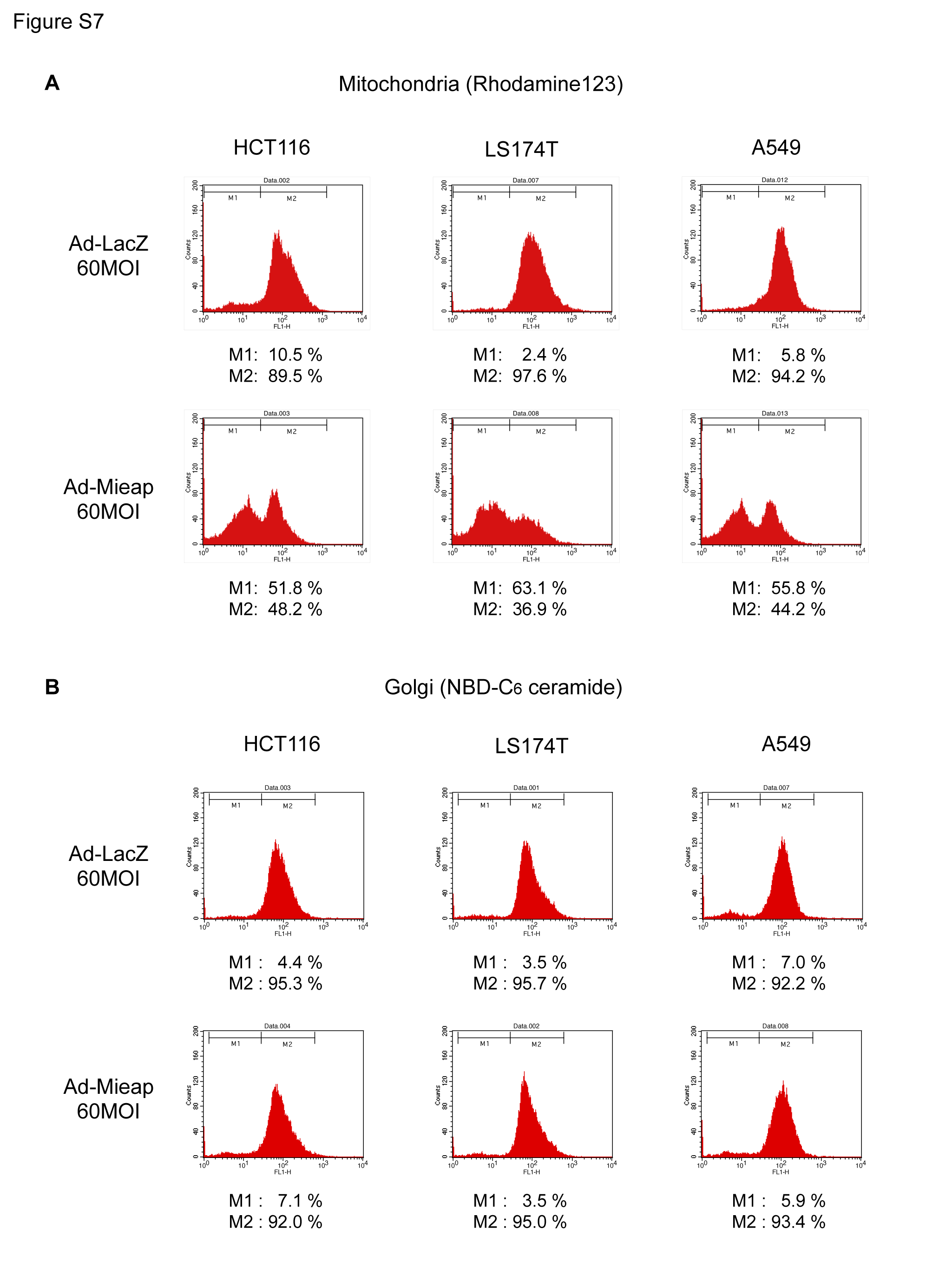

Supplement: Figure S7 — MIV specifically eats and degrades mitochondria. (A) (B) HCT116, LS174T, and A549 cells were infected with Ad-Mieap or Ad-LacZ at an MOI of 60. After 24 h, mitochondria or Golgi were stained by Rhodamine123 (A) or NBD-C6-ceramide (B), respectively. The signals of mitochondria or Golgi were analyzed by fluorescence activated cell sorting (FACS). (TIF) [file pone.0016060.s007.tif]

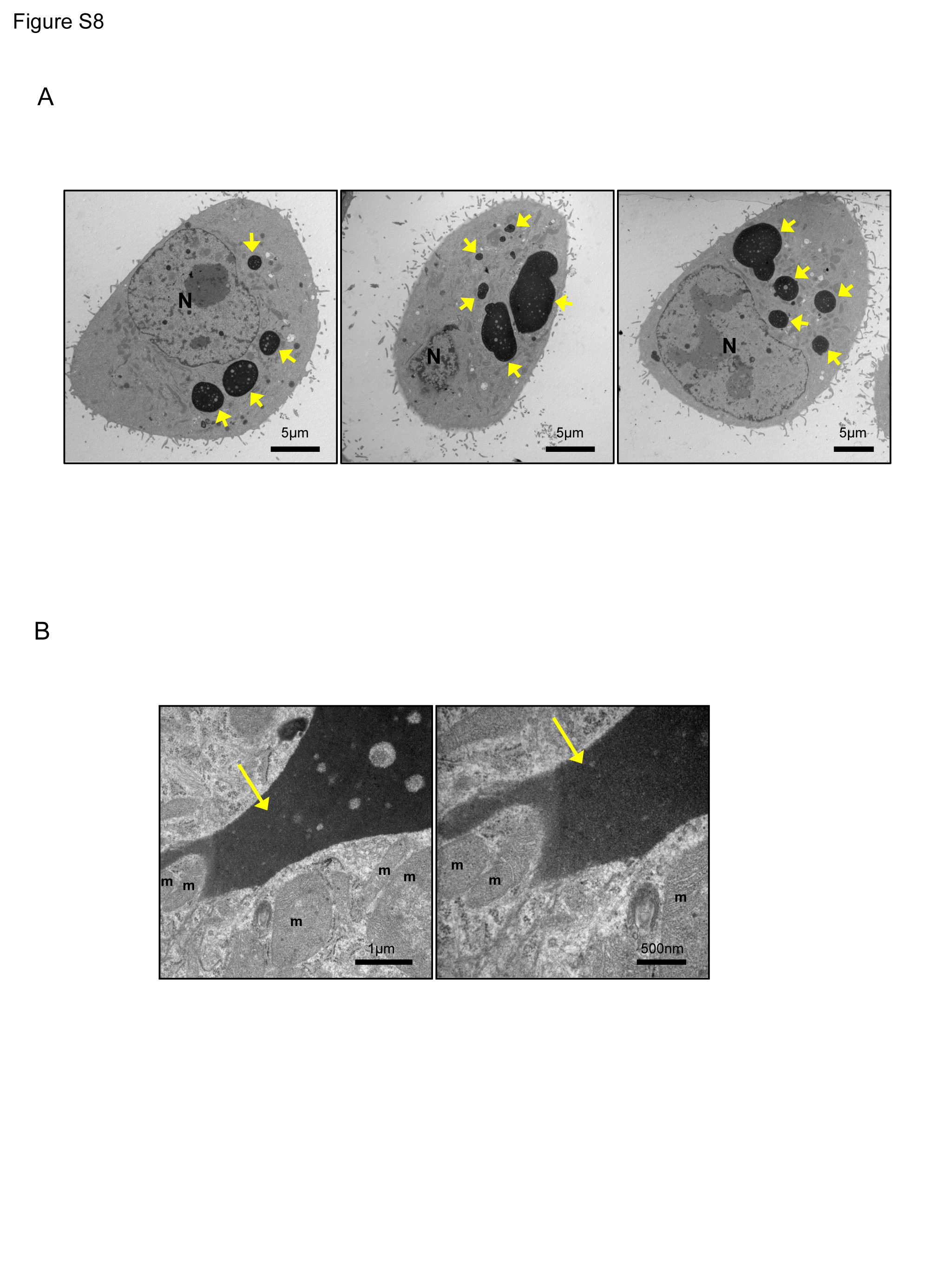

Supplement: Figure S8 — Electron microscopic analysis on the MIVs. (A) Consistent with the data in other experiments (Figure 5A–E), the MIVs were detected by electron microscopic analysis as various sizes of extremely-dense and round structures. Yellow arrows indicate MIVs. N: nucleus Scale bar = 5 µm (B) MIV may engulf mitochondria in a manner that is similar to yeast selective microautophagy of mitochondria. Yellow arrow indicates MIV. m: mitochondria Scale bar = 1 µm or 500 nm. (TIF) [file pone.0016060.s008.tif]

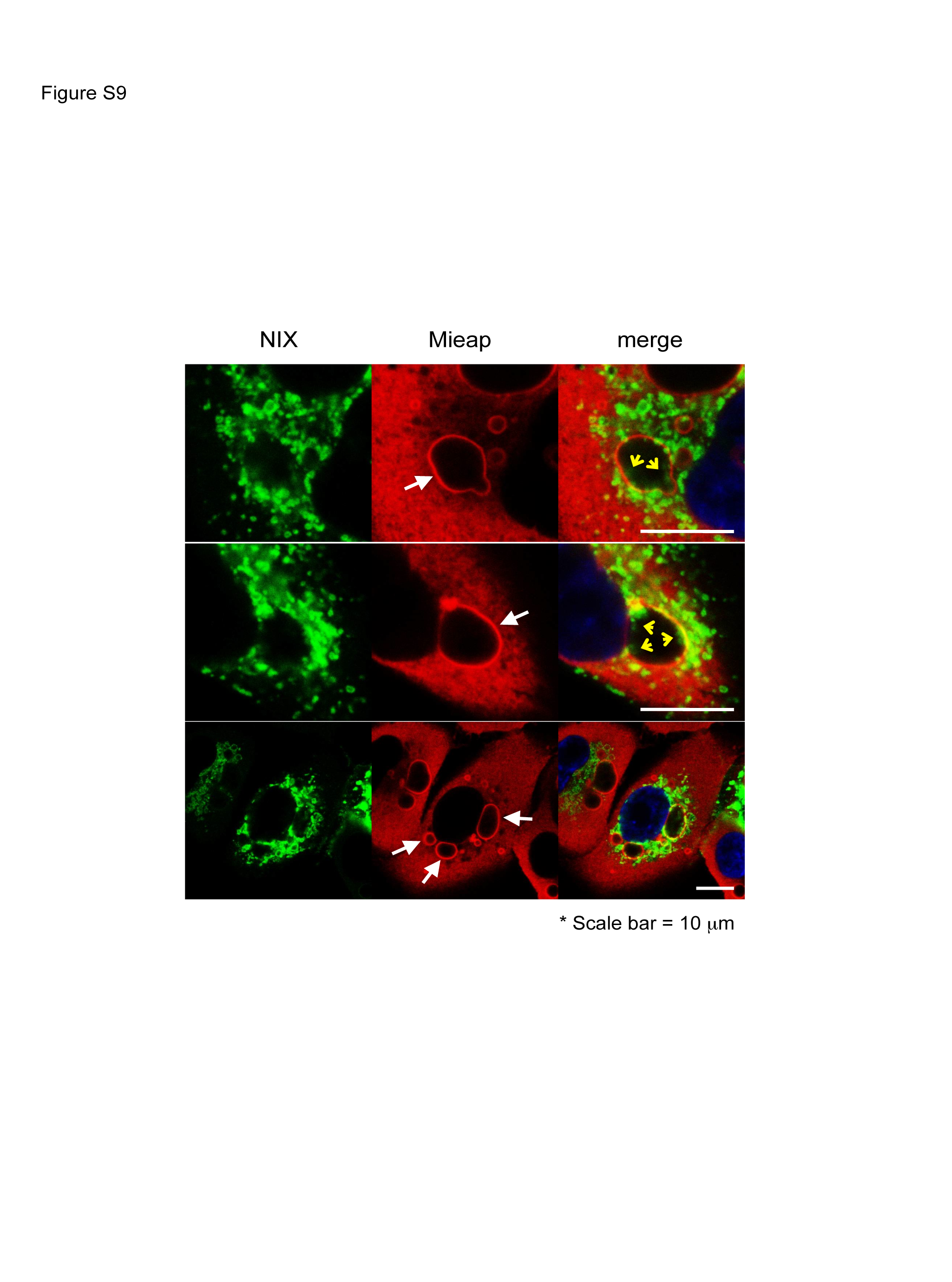

Supplement: Figure S9 — A mitochondrial outer membrane protein NIX is degraded by MIV. In order to examine the relationship between NIX and MIV, the IF experiment was carried out. A549 cells were transfected by the plasmid designed to express the N-FLAG-tagged NIX, and 2 h after the transfection, the cells were infected with adenovirus vector designed to express Mieap at an MOI of 5. 36 h after the infection, the cells were subjected to IF experiment with anti-FLAG antibody (NIX: green), and anti-Mieap antibody (Mieap: red). The representative images were shown. White arrows indicate MIVs. Yellow arrows indicate the degradation of NIX within MIV. Scale bar = 10 µm. (TIF) [file pone.0016060.s009.tif]

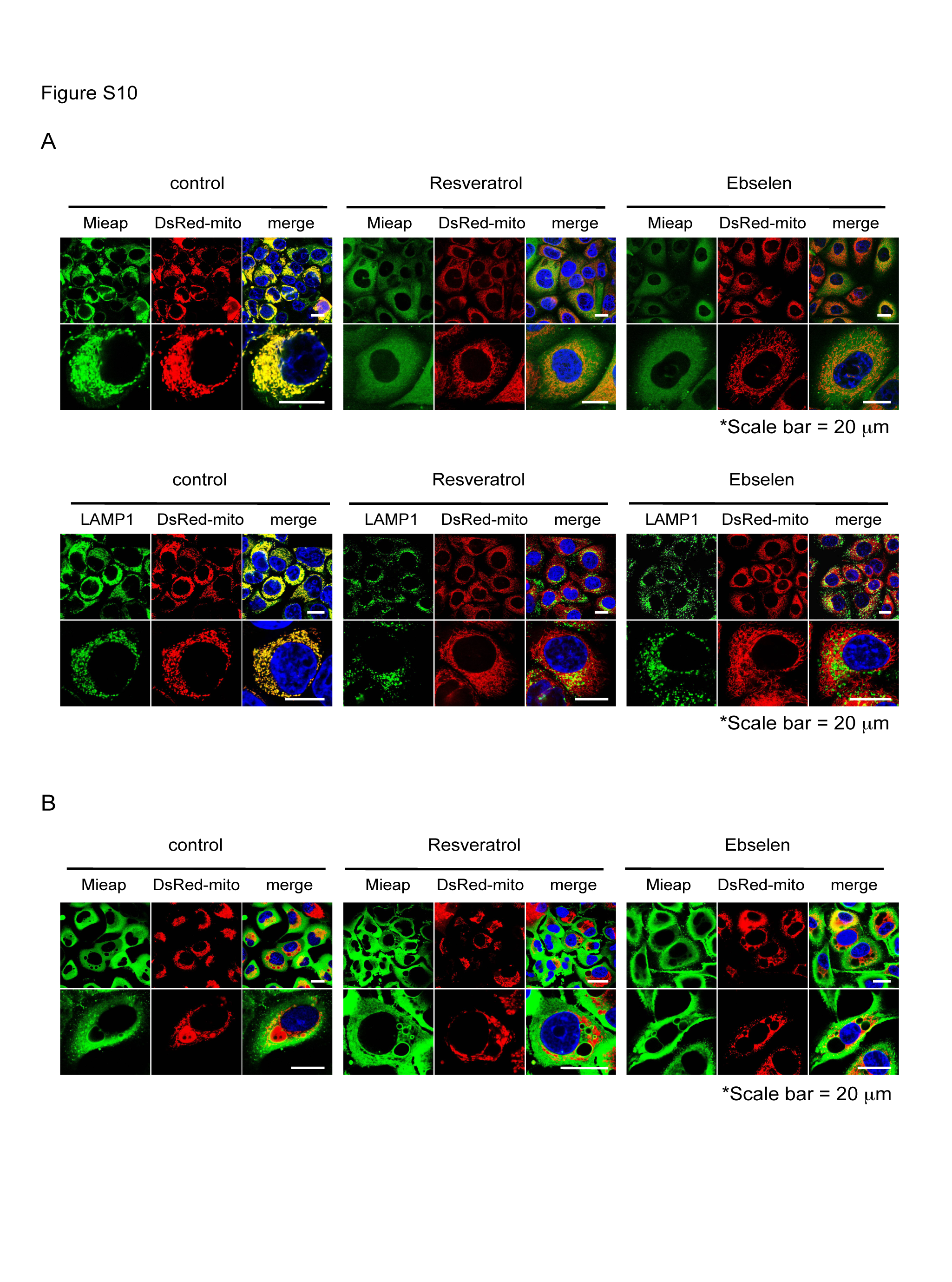

Supplement: Figure S10 — Resveratrol and Ebselen inhibit MALM and uptake of mitochondria by MIV. To examine the effect of resveratrol and ebselen on MALM, A549 cells were infected with Ad-Mieap at an MOI of 5 and Ad-DsRed-Mito at an MOI of 30. 24 h after the infection, the cells were irradiated by γ ray, and 2 h after IR, 25 µM resveratrol or 10 µM Ebselen was added to the culture media. On day 3 after IR, the cells were subjected to IF experiment. To examine the effect of resveratrol and ebselen on MIV, A549 cells were infected with Ad-Mieap at an MOI of 60 and Ad-DsRed-Mito at an MOI of 30. 2 h after the infection, 25 µM resveratrol or 10 µM Ebselen was added to the culture media. 24 h after the infection, the cells were subjected to IF experiment. The IF experiment was carried out with rabbit polyclonal anti-Mieap antibody (Mieap: green), mouse monoclonal anti-LAMP1 antibody (LAMP1: green), and DsRed-Mito (mitochondria: red). Scale bar = 20 µm. (TIF) [file pone.0016060.s010.tif]

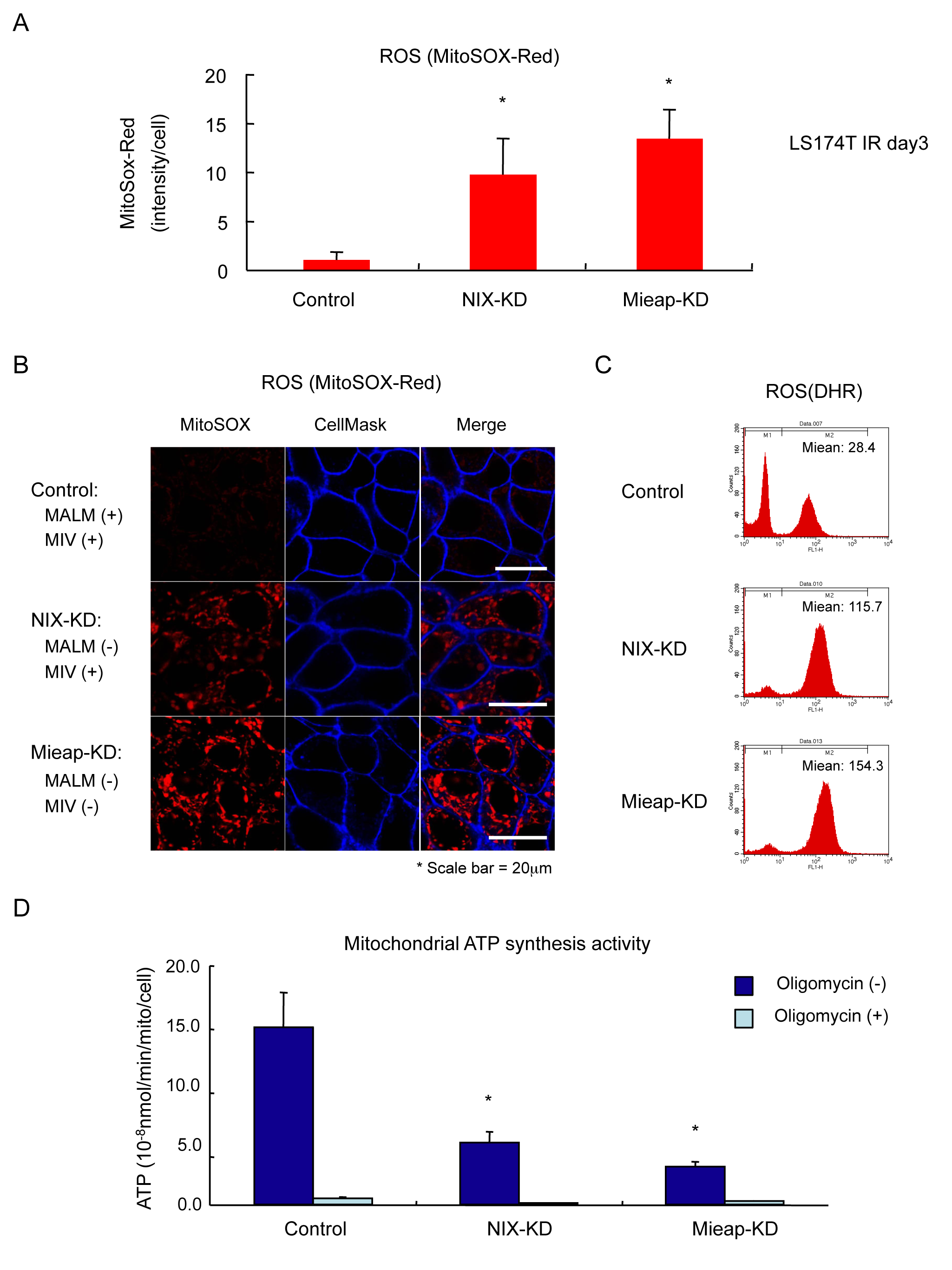

Supplement: Figure S11 — MIV eliminates unhealthy mitochondria. (A) (B) (C) Mitochondrial ROS level. The control, NIX-KD, and Mieap-KD cells of LS174T were irradiated by γ ray, and the ROS generated by mitochondria in the cells were analyzed by MitoSox-Red on day 3 after IR. (A) Quantitative analysis of ROS was carried out in 300–400 cells (lower panel). Average intensities of ROS per cell are shown with error bars indicating 1 SD. p<0.01 (*) was considered statistically significant. (B) The representative images are shown. Scale bar = 20 µm (C) The ROS level was analyzed by FACS with dihydrorhodamine (DHR). (D) ATP synthesis activity by the mitochondria. The cells were subjected to ATP synthesis assay on day 3 after IR. Oligomycin, an inihibitor of mitochondrial oxidative phosphorylation, was used in the assay in order to detect non-mitochondrial ATP synthesis activity. The average activities of ATP synthesis are shown with error bars indicating 1 SD. p<0.01 (*) was considered statistically significant. (TIF) [file pone.0016060.s011.tif]

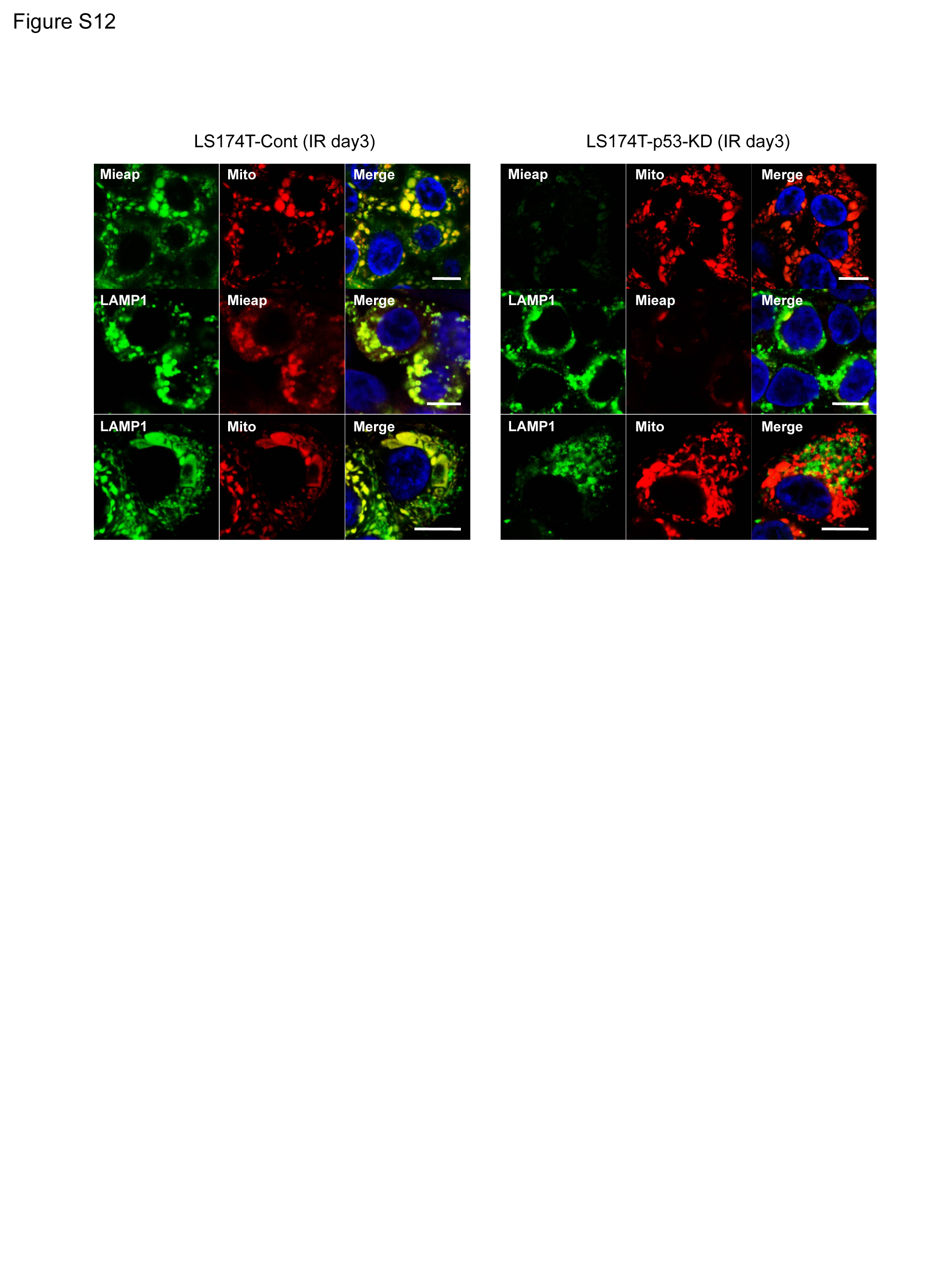

Supplement: Figure S12 — p53 controls mitochondrial quality. p53 regulates MALM. The cont and p53-KD cells of LS174T were subjected to IF experiment on day 3 after IR. Mieap protein was stained with polyclonal rabbit anti-Mieap antibody (Mieap: green or red). Lysosomes were stained with mouse monoclonal anti-LAMP1 antibody (LAMP1: green). Mitochondria were indicated by the DsRed-mito protein signal (Mito: red). Scale bar = 10 µm. (TIF) [file pone.0016060.s012.tif]

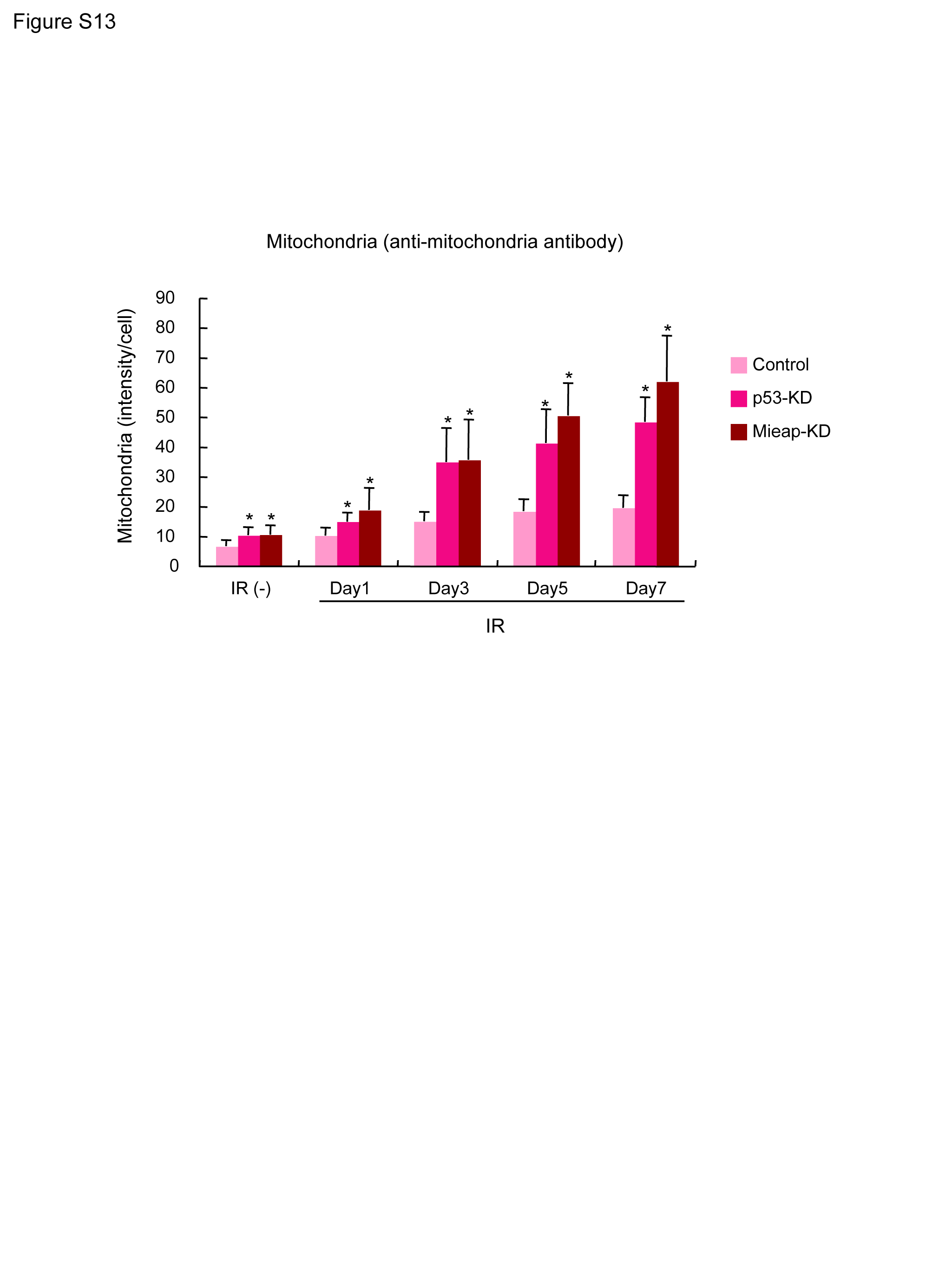

Supplement: Figure S13 — The mitochondrial intensity increases in p53-defective cells. The cells were irradiated by γ ray, and the mitochondrial intensity was analyzed by the signal of mouse monoclonal anti-human mitochondria antibody (Leinco Technology, MO, USA: clone AE1) at the indicated times. Quantitative analysis of mitochondrial intensity was carried out in 300–400 cells. Average intensities of mitochondria per cell are shown with error bars indicating 1 standard deviation (SD; left panel). p<0.01 (*) was considered statistically significant. (TIF) [file pone.0016060.s013.tif]
